# Unraveling risk factors and transcriptomic signatures in liver cancer progression and mortality through machine learning and bioinformatics

**DOI:** 10.1093/bfgp/elaf019

**Published:** 2026-01-09

**Authors:** Tania Akter Asa, Md Ali Hossain, Md Shahjahan Ali, Md Zulfiker Mahmud, A K M Azad, Mohammad Zahidur Rahman, Mohammad Ali Moni

**Affiliations:** NanoBio Technology Center, Daffodil International University, Birulia, Savar, Dhaka-1216, Bangladesh; Dept of Computer Science and Engineering, Jagannath University, 9-10 Chittaranjan Avenue, Sadarghat, Dhaka-1100, Bangladesh; NanoBio Technology Center, Daffodil International University, Birulia, Savar, Dhaka-1216, Bangladesh; Dept of Computer Science and Engineering, Jahangirnagar University, Savar, Dhaka-1342, Bangladesh; Dept of Electrical and Electronic Engineering, Islamic University, Kushita-7003, Bangladesh; Dept of Computer Science and Engineering, Jagannath University, 9-10 Chittaranjan Avenue, Sadarghat, Dhaka-1100, Bangladesh; Department of Mathematics and Statistics, Faculty of Science, Imam Mohammad Ibn Saud Islamic University (IMSIU), Riyadh 13318, Kingdom of Saudi Arabia; Dept of Computer Science and Engineering, Jahangirnagar University, Savar, Dhaka-1342, Bangladesh; AI and Digital Health Technology, Artificial Intelligence & Cyber Futures Centre, Charles Sturt University, Bathurst, NSW 2795, Australia; AI and Digital Health Technology, Rural Health Research Institute, Charles Sturt University, Orange, NSW 2800, Australia

**Keywords:** liver cancer, risk factors, clinical factors, gene expression, COX PH model, survival analysis, RNA-seq, molecular pathways

## Abstract

Liver cancer (LC) is the second leading cause of cancer-related deaths globally, yet the molecular mechanisms linking its progression with associated risk factors (RFs) remain poorly understood. To address this, we developed an integrative multi-stage framework combining bioinformatics, machine learning-based feature selection, survival modeling, and network analysis to identify robust biomarkers and pathways involved in LC progression. Unlike conventional biomarker discovery approaches, our strategy integrates multi-cohort transcriptomic and clinical datasets, enhancing robustness and reliability of findings. Initially, differentially expressed genes were identified from three Gene Expression Omnibus datasets for LC and its RFs. Next, using shared biomarkers, we constructed a gene-disease association (diseasome) network, revealing 230 unique genes, including 126 shared between LC and liver cirrhosis. Subsequently, RNA-seq and clinical data from The Cancer Genome Atlas (TCGA) were analyzed through combined and multivariate Cox survival models, identifying 70 prognostic genes. Among these, we identified RGS5, SULT1C2, CSM3, and CXCL14 as consistent survival-associated markers. Functional investigation of the 70 genes using enrichment and protein–protein interaction networks uncovered ten hub genes involved in key oncogenic pathways, including Oocyte meiosis, Lysine degradation and cell cycle regulation. These findings were further validated through literature and expression-level analysis. Additionally, an independent survival analysis using the full TCGA transcriptomic dataset identified 76 significant genes, with 18 overlapping the risk-associated gene set, reinforcing their prognostic value. Overall, this study demonstrates the potential of an integrative computational approach to uncover meaningful biomarkers and pathways in LC, offering valuable insights for future clinical and therapeutic strategies.

## Introduction

Liver cancer (LC) is a leading cause of cancer-related mortality globally, ranking as the fifth most common cancer and the second leading cause of cancer deaths worldwide [1–3]. Men are at higher risk of developing LC, with a male-to-female ratio of 2.8:1 [3]. In 2018, an estimated 841,000 new LC cases were diagnosed, with 782,000 deaths [2]. LC is particularly prevalent in Asia and Africa and is often linked to cirrhosis (CHRS), hepatitis B virus (HTBV), and hepatitis C (HTCV) infections [3–5]. Other risk factors include ageing (AG), Type 2 diabetes (T2D), alcohol consumption (ALC), obesity (OB), and smoking (SMK) [3, 6].

LC is classified into primary and secondary types, with hepatocellular carcinoma (HCC) accounting for over 80% of primary cases [3]. Primary LC originates in the liver, whereas secondary LC develops in other body organs and spreads to the liver through metastasis. This type of LC is of 10 linked to cancers such as colorectal, breast, pancreatic, ovarian, and lung cancers. LC involves the presence of malignant tumors within or on the liver [3]. Despite extensive research, the molecular mechanisms underlying LC progression remain poorly understood. Identifying biomarkers for early diagnosis and better treatment strategies has become a major area of focus.

So, to explore the actual reason for the development of LC, the association of LC with its RFs needs to be studied more. A number of genetic studies have been carried out to identify biomarkers of LC [3, 7–11]. For instance, Chidambaranathan *et al.* reviewed the links between LC and its RFs [3]; however, their study lacked a comprehensive understanding of the disease’s molecular progression. Singh *et al.* [10] explored modular co-expressed genes across different RFs and histological grades of HCC, identifying key pathways involved in metabolism, immune regulation, and cell signaling. While their findings highlighted the variability of molecular mechanisms across different RFs, they did not integrate survival analysis or validate their results using independent datasets. However, their study lacked a comprehensive gene-network analysis to systematically link LC to its RFs. Similarly, Talubo *et al.* [11] emphasized the complexity of HCC pathogenesis and the necessity of multi-marker-based biomarker identification but did not investigate the gene-disease relationships using a network-based approach. These gaps highlight the need for an integrated multi-omics framework that not only identifies key genes but also explores their clinical relevance in LC progression and patient survival.

In this study, we applied bioinformatics and machine learning approaches to identify LC risk genes associated with key RFs, including CHRS, HTCV, HTBV, AG, ALC, T2D, OB, and SMK. To achieve this, we used high-throughput transcriptomics datasets (microarray and RNA Seq) and employed a network-based multi-omics approach to analyze protein–protein interaction (PPI) sub-networks, gene ontologies, and molecular pathways, revealing the genetic influence of these factors on LC progression. We first identified differentially expressed genes (DEGs) for LC and its RFs, followed by detecting common genes shared between LC and its eight RFs. A gene-disease association network was then constructed to map the molecular links between LC and its comorbidities.

To evaluate the prognostic relevance of the identified genes, we integrated clinical data from The Cancer Genome Atlas (TCGA) with corresponding mRNA expression profiles, enabling a comprehensive survival analysis. Using Cox Proportional Hazard (PH) regression models, we conducted univariate, multivariate, and combined analyses to assess the impact of both clinical factors and gene expression on LC patient outcomes. Furthermore, we validated the expression of common genes with TCGA-derived transcriptomic data. Finally, PPI network, common pathway and GO ontology analyses were performed on identified genes obtained from the two analyses (combined and multivariate) to identify hub proteins and significant pathways that are implicated with the comorbidity of LC with its eight risk factors. This approach allowed us to pinpoint key molecular interactions that drive LC progression in the context of its eight major risk factors.

Through this comprehensive computational framework, our study not only enhances our understanding of LC pathogenesis but also provides potential biomarkers for improved diagnosis and prognosis. The integration of multi-omics data with clinical survival analysis represents a significant step toward identifying molecular targets for therapeutic interventions in LC patients.

## Materials and methods

The Materials and Methods section outlines the datasets, preprocessing steps, and analytical techniques used in this study. We describe the gene expression analysis, survival modeling approaches, and machine learning techniques applied to identify prognostic biomarkers for LC.

### Data collection

For this research work, datasets were collected from the National Center for Biotechnology Information (NCBI) Gene Expression Omnibus (GEO) as well as dataset from the TCGA genome data analysis center.

#### Data acquisition from NCBI

To identify the molecular association of different risk factors (RF) of LC with LC, Gene expression microarray datasets of LC and its risk factors Diseases (RFDs) were collected from NCBI (GEO dataset) and analysed. The accession numbers of the 10 different datasets are: GSE134921, GSE101728, GSE101685 for LC, GSE102451(CHRS), GSE110312(HTCV), GSE135501 (HTBV), GSE674 (AG), GSE52553(ALC), GSE23343(T2D), GSE48964(OB), and GSE4806(SM). The descriptions of the datasets used in this study are shown in Table 1. The LC dataset (GSE134921) contains 21 HCC samples where 10 MRSlow and 11 MRShigh samples. Another LC dataset (GSE101728) contains mRNA data of seven pairs of HCC and matched adjacent tumor-free tissues. The third LC dataset GSE101685 contains mRNA data of 8 normal tissue and 24 hepatocellular carcinoma patients. The AG dataset (GSE674) is obtained by analyzing gene expression in vastus lateralis skeletal muscle biopsies in healthy young (20–29-year-old) and older (65–71 year old) human [12]. The dataset for alcohol consumption (AC) (GSE52553) is obtained from ethanol treatment of lymphoblastoid cell lines from alcoholics and non-alcoholics causes many subtle changes in gene expression [13]. The diabetic dataset (GSE23343) is an Affymetrix Human Genome data from the human liver with or without T2D [14]. The OB dataset (GSE48964) is obtained by expression data from Adipose Stem Cells from morbidly obese and non-obese individuals [15]. The SMK dataset (GSE4806) is obtained from gene expression profiles of T-lymphocyte smokers and non-smokers [16]. The CHRS dataset (GSE102451) is obtained by analyzing gene expression in Cirrhosis patients and hepatocellular carcinoma patients. The HTC Virus dataset GSE110312 is Affymetrix Human Genome data from Huh7-MAVSR cell that were infected with cell culture HCV at an MOI of 5 for 12, 24, 48, 72 hours and uninfected Huh7- MAVSR cells that were similarly cultured for 72 h, used as mock infection control. The HTB Virus dataset GSE135501 is Affymetrix Human Genome data from Chronic hepatitis B patients and healthy controls.

**Table 1:** Dataset descriptions of used LC and LDIs diseases

Diseases Name	GSE Number	Case	Control
Liver Cancer	GSE134921	11 MRS high	10 MRS low
Liver Cancer	GSE101728	Liver cancer tissues: 7	Non-tumor tissues: 7
Liver Cancer	GSE101685	Liver cancer patients: 24	Non-tumor tissues: 8
Cirrhotic (CHRS)	GSE102451	Hepatocellular carcinoma from cirrhotic liver: 27	Non-tumor cirrhotic tissue: 30
HTCV	GSE110312	Case: 4	Healthy controls: 1
HTBV	GSE135501	Case: 40	Healthy controls: 14
Aging	GSE674	Older (65–71-year-old) humans: 19	Vastus lateralis skeletal muscle biopsies in young: 30
Alcohol Consumption	GSE52553	Alcoholics: 21	Non-alcoholics: 21
Type 2 Diabetes (T2D)	GSE23343	Patients with T2D: 10	Healthy subjects: 7
Obese	GSE48964	Morbidly obese: 3	Non-obese individuals: 3
Smoking	GSE4806	Smokers: 3	Non-smokers: 3

#### Data acquisition from TCGA

The Cancer Genome Atlas (TCGA) genome data analysis center. (http://gdac.broadinstitute.org/) is an interactive data system for researchers to search, upload, download, and analyse cancer genomic data [17–19] and we collected the mRNAseq data of LC for this study from this source. Since our goal was to explore a particular point of interest, survival analysis of LC on clinical and genetic factors, we retrieved the anonymized clinical data and RNAseq data for LC (Liver Hepatocellular Carcinoma [TCGA, Provisional]) from the cBioPortal [19, 20].

In our clinical dataset, there were 442 cases with 84 features and there were 373 cases that had mRNAseq gene expression data with 20,440 genes in the RNAseq dataset. We selected eight clinical factors (Race Category, Diagnosis Age, Sex, *Histologic* − *Grade*, Cancer stage, Person Neoplasm Status, Days to Last Follow up and Censor Status) along with significant genes common with LC and its risk factors. We investigated a single outcome variable, namely LC-specific survival using these data. We matched patient ID in both clinical and RNAseq datasets and identified 365 patients with data available for both and took eight clinical variables given above. The diagnosis of age and Cancer Type were collected from pathology reporting. Patient survival data were taken from the overall number of months of patient survival.

**Figure 1:**
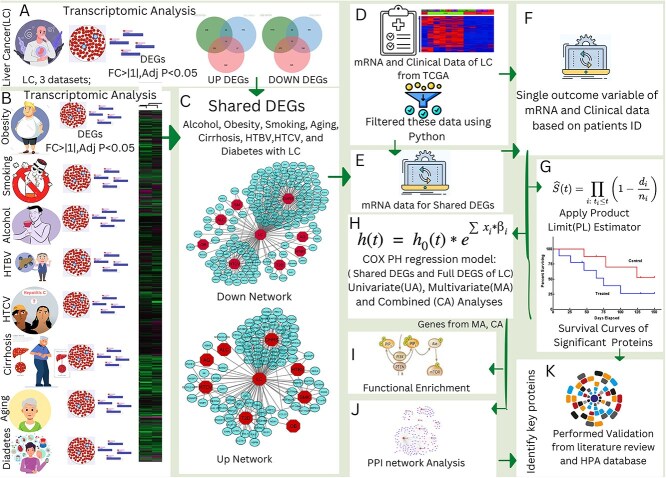
The multi-stage analysis methodology was used in this study. (A, B) Common differentially expressed genes were identified among LC and its eight risk factors (Liver Cirrhosis, HTBV, HTCV, Ageing, Type 2 diabetes, heavy alcoholing, OB, and smoking). (C) Gene-Disease association network of LC with its eight risk factors was built. (D) Filter mRNA and clinical data of LC. (E, F) Identified the mRNA of shared genes of LC with its risk factors by comparing with the mRNA data of LC obtained from TCGA and then combined mRNA and clinical data by using patient IDs. (G) Apply product limit estimator for the survival curves of significant genes. (H) Performed univariate, multivariate, and combined analyses on the clinical and mRNA data to identify genes and clinical factors associated with LC patient survival. (I, J) PPI network, common pathway and GO ontology analyses were performed on identified genes obtained from the 2 analyses (combined and multivariate analyses) to identify hub proteins, and significant pathways that are implicated with comorbidity of LC with its 8 risk factors. (K) The hub genes were validated using literature reviews and data from HPA database.

The work steps of this research are shown in Fig. 1. The workflow of this study follows a comprehensive multi-stage analysis to explore the molecular link between liver cancer (LC) and its associated risk factors. Initially, gene expression data for LC and its eight risk factors were obtained from publicly available databases, including NCBI GEO and TCGA. Subsequently, common DEGs between LC and each risk factor were identified, focusing on genes that exhibited consistent expression patterns across both conditions. Next, a gene-disease association network (diseasome) was constructed, leveraging Cytoscape and applying topological and neighborhood-based benchmarking methods to reveal key molecular interactions. Following this, mRNA expression data were integrated with clinical data, matched by patient IDs, to ensure data coherence. Thereafter, Kaplan–Meier survival curves were generated, and combined, multivariate, and univariate Cox regression analyses were applied to assess the impact of genes and clinical factors on LC survival. Additionally, PPI network analysis was performed to identify hub proteins linked to significant DEGs, followed by pathway enrichment and Gene Ontology (GO) analysis to uncover critical biological processes. Finally, findings were validated through a thorough literature review and corroborated with data from the Human Protein Atlas (HPA). Additionally, an independent survival analysis using the full TCGA transcriptomic dataset identified 76 significant genes, with 18 overlapping the risk associated gene set, reinforcing their clinical relevance.

#### DEGs analysis and diseasome network construction

We first identified DEGs in three LC datasets and eight risk factor datasets using the limma _package in R. A threshold of log FC_ ≥ 1 and an adjusted *P*-value < 0.05 was applied, where the adjusted values were computed using the Benjamini-Hochberg (BH) False Discovery Rate correction to control for multiple hypothesis testing and reduce the likelihood of false positives.

We then focused on the DEGs of LC that were common across two sets of LC datasets and present in the DEGs of each. We identified the concordant genes that exhibited consistent expression patterns in both LC and its eight risk factors (CHRS, HTCV, HTBV, AG, ALC, T2D, OB, and SM). To construct the diseasome network, Topological and Neighbourhood-based benchmark methods [21, 22] were used, which were better suited for this network. We then constructed the diseasome network linking LC with its risk factors using Cytoscape [23].

#### Gene expression analysis of the TCGA dataset

Getting common genes of LC and its risk factors from the above operation, we identified the mRNA and clinical data of these common genes by comparing them with the mRNA data of LC obtained from TCGA.

In our analysis, we applied a z-score transformation to standardize gene expression values, considering a reference population of either all diploid tumors or, when available, normal adjacent tissues. The z-score indicates the number of standard deviations a gene’s expression deviates from the reference mean. To enhance robustness, we adopted a more statistically stringent threshold of |*z*| ≥ ±1.96, aligned with a confidence level 95% in a standard normal distribution.

We computed z-scores for RNAseq data using the following formula:


(1) \begin{align*} \mathrm{z}=&(\mathrm{Expression}\kern0.17em \mathrm{for}\kern0.17em \mathrm{gene}\;\mathrm{X}\;\mathrm{in}\kern0.17em \mathrm{tumor}\;\mathrm{A}-\mathrm{Mean}\kern0.17em \mathrm{expression}\kern0.17em \mathrm{for}\notag\\&\kern0.17em \mathrm{gene}\mathrm{X}\;\mathrm{in}\kern0.17em \mathrm{normal})/\mathrm{Standard}\kern0.17em \mathrm{deviation}\kern0.17em \mathrm{of}\kern0.17em \mathrm{expression}\kern0.17em \mathrm{for}\notag\\& \mathrm{gene}\;\mathrm{X}\;\mathrm{in}\kern0.17em \mathrm{reference}\kern0.17em \mathrm{population} \end{align*}

We thus used the z-score values to define samples with “altered” (Over-express or Under express) and “normal” (unaltered) expression of a gene. A gene was considered altered if its z-score was equal to or greater than 2, ensuring statistical significance in deviation. This classification is formally defined as follows:


$$ {\displaystyle \begin{array}{c}z\ge 2=> altered\\{}z<2=> normal\end{array}} $$

### Machine learning-based identification of key proteins and survival prediction

The following analyses were performed based on the survival of patients with LC. At first, the product-limit (PL) estimator was used for calculating the estimation of survival function, then the log-rank test was performed to identify statistically significant differences between two groups (patients with altered gene expression and patients with unaltered gene expression) and next Cox PHs regression models were used to identify the significance of genes and clinical factors. Benjamini-Hochberg correction was applied to adjust for multiple testing. We employed important clinical variables that affect LC obtained literature review and significant genes of LC common with its risk factor diseases. The Z-score value of each gene was converted to altered or normal categories based on the threshold value of the Z-score (*z* > 2) as described in the data section. Next, we performed combined analyses (taking all genes and clinical variables simultaneously), multivariate (taking all genes simultaneously) and univariate (taking all genes individually). Survival analysis is a statistical analysis used for estimating the expected duration of time until one or more events happen, such as failure in mechanical systems and death in cancer. We used a PL estimator (a non-parametric technique) for the estimation of the survival function. In short PL estimator of the survival function can be defined as follows:


$$ S\hat{\mkern6mu} \left({\mathrm{t}}_{\mathrm{b}}\right)={\prod}_{\mathrm{a}=1}^{\mathrm{b}}\left(1-{\mathrm{d}}_{\mathrm{b}}/{\mathrm{n}}_{\mathrm{b}}\right)$$

Here,

Ŝ (*t_b_*): estimated survival function at time *t_b,_*

*d_b_*: Number of events occurred at *t_b_*,

*n_b_*: Number of subjects available at *t_b_*.

After the estimation of survival function using PL, Log-rank tests were used in altered *versus* unaltered groups in the context of gene expression to identify the most significant genes.

The null hypothesizes are given below:

*H*_0_: Survival functions for patients with altered gene expression and the patients with unaltered gene expression are the same.

*H_A_*: Survival functions for these two groups are not the same. These functions can be written Symbolically as


$$ {\displaystyle \begin{array}{c}{H}_0:{S}_{altered}(t)={S}_{not\ altered}(t)\\{}{H}_A:{S}_{altered}(t)\ne{S}_{not\ altered}(t)\end{array}} $$

For survival analysis, Cox PH regression is the most popular regression technique which is used to associate one or several risk factors or exposures, considered simultaneously, to survival time. Applying the Cox PH regression model, we first performed a **combined analysis** by incorporating all selected clinical factors together with the 215 genes, modeling the hazards of Liver Hepatocellular Carcinoma (TCGA, Provisional) under an undetermined baseline hazard function with an exponential form of covariates. Next, we carried out a **multivariate analysis**, considering all 215 genes simultaneously. Finally, we conducted a **univariate analysis**, where each gene was evaluated separately to identify its individual prognostic significance. Mathematically we can write the model as follows:


$$ h\left(t|{X}_i\right)={h}_0(t)\mathit{\exp}\left({\beta}^T{X}_i\right) $$

Where, *h*(*t*|*X*): the hazard function conditioned on a subject i with covariate information given as the vector *x_i_*, *h*_0_(*t*) is the baseline hazard function which is independent of covariate information, β: represents a vector of regression coefficients to the covariates correspondingly.

### Independent survival analysis using full transcriptomic dataset

To address potential limitations of using only risk-associated genes for survival prediction, we performed an independent survival analysis using the full transcriptomic dataset from The Cancer Genome Atlas (TCGA-LIHC). Initially, 1045 DEGs were identified from three GEO datasets related to liver cancer. Expression data for 1005 of these genes were retrieved from the TCGA RNA-seq dataset. After preprocessing and filtering based on data completeness and variability, 967 genes were retained for survival analysis. We employed univariate Cox proportional hazards regression to evaluate the association between gene expression and overall survival. Additionally, multivariate and combined Cox regression was performed. This approach enabled the identification of robust transcriptomic biomarkers associated with patient survival, independent of risk factor filtering.

### Functional enrichment of differentially expressed genes obtained from two analyses

To get further insight into the molecular pathways of LC that overlap with CHRS, HTCV, HTBV, AG, ALC, T2D, OB, and SM, by genes obtained from two analyses (combined and multivariate), we performed pathway and gene ontology analysis using ClusterProfiler R package for these analyses [24, 25]. For getting significant enrichment results, we considered *adjusted* − *P* < .05 as the threshold *P*-value.

### Proteomic signatures: hub proteins from protein–protein interaction analysis

We also generated a PPI network around significant DEGs, found from the two analyses (combined and multivariate analysis) of LC by using a web-based visualization software NetworkAnalyst [26, 27] and STRING database [26, 28]. After that, topological analysis was applied to identify highly connected proteins (*i.e.* hub proteins) by employing the degree metrics and betweenness Centrality [29].

## Results

### Identification of differentially expressed genes in liver cancer

To investigate transcriptomic alterations in liver cancer (LC), we analyzed three microarray datasets from the GEO repository (GSE101728, GSE134921, and GSE101685) based on predefined selection criteria. These DEGs were obtained using the limma package with empirical Bayes moderation and BH-adjusted p-values_._ The significance threshold was set to log FC ≥ 1 and an adjusted *P*-value < 0.05.

**Figure 2:**
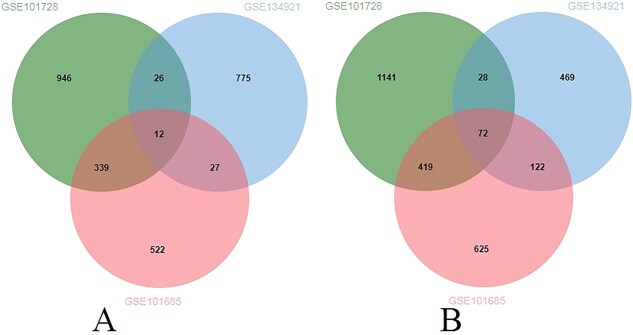
This figure illustrates the common DEGs among all three transcriptomics datasets using Venn diagram. (A) shows the upregulated genes and (B) shows the downregulated genes.

After filtering for statistical significance, we identified 2983 DEGs (1323 upregulated, 1660 downregulated) in GSE101728 (see Supplementary File 1), 1531 DEGs (840 upregulated, 691 downregulated) in GSE134921 (see Supplementary File 2), and 2100 DEGs (900 upregulated, 1200 downregulated) in GSE101685 (see Supplementary File 3). To ensure robustness, we considered only those DEGs that were common between at least two datasets, yielding a final set of 1045 DEGs (404 upregulated, 641 downregulated) for downstream analysis. A Venn diagram was used to illustrate the overlap of common DEGs across the three datasets (Fig. 2A and B).

### Cross-disease comparative analysis of liver cancer and its risk factors

**Figure 3:**
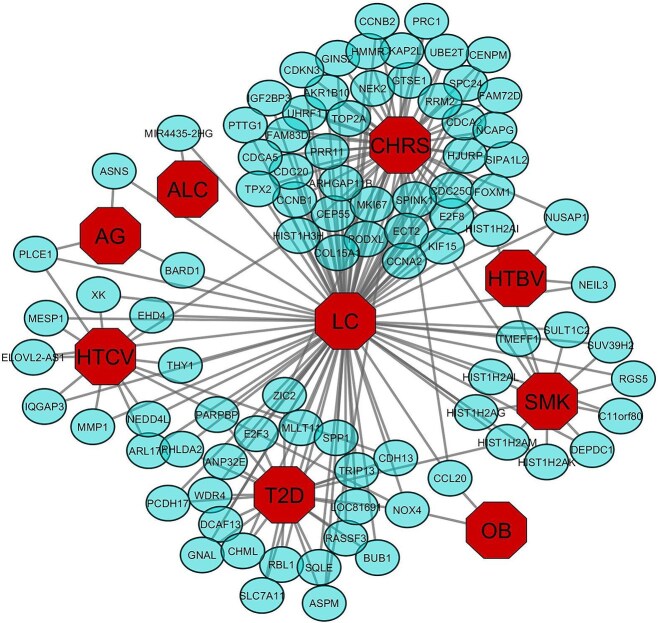
Gene-diseasome network of LC with CHRS, HTCV, HTBV, AG, ALC, T2D, OB, and SMK for upregulated genes.

**Figure 4:**
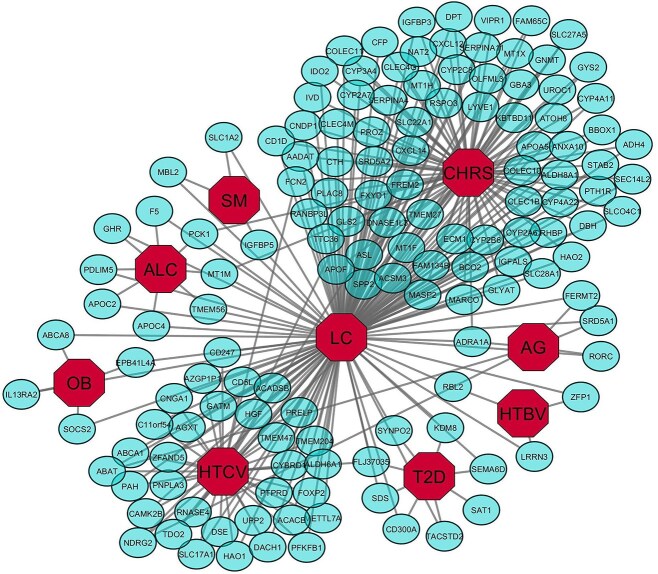
Gene-diseasome network of LC with CHRS, HTCV, HTBV, AG, ALC, T2D, OB, and SMK for down regulated genes.

Given the multifactorial nature of LC, we explored its molecular associations with eight known risk factors: CHRS, HTCV, HTBV, AG, ALC, T2D, OB, and SMK. Transcriptomic data for each risk factor were analyzed separately to identify DEGs. We identified 240 DEGs in CHRS, 2224 in HTCV, 290 in HTBV, 322 in AG, 260 in ALC, 146 in OB, 394 in SMK, and 1385 in T2D. A cross-comparative analysis between LC and each risk factor revealed common DEGs, suggesting potential molecular links. Specifically, LC shared 46, 13, 2, 3, 1, 23, 2, and 12 significantly up-regulated genes with CHRS, HTCV, HTBV, AG, ALC, T2D, OB, and SMK, respectively (Fig. 3). Similarly, 80, 39, 3, 6, 8, 8, 4, and 3 significantly down-regulated genes were shared with these risk factors (Fig. 4).

Further analysis highlighted additional genes involved in multiple risk factors, underscoring their relevance in LC pathophysiology.

### Diseasome network construction

To elucidate molecular interactions between LC and its associated risk factors, we constructed a diseasome network (gene-disease association network) using Cytoscape. The network was based on shared DEGs between LC and each risk factor, revealing key molecular interconnections. Topological and neighborhood-based benchmarking methods were used to assess network robustness and biological significance (Figs 3 and 4). Notably, 6 significant genes (*FCN2, FREM2, GLS2, ACSM3, FAM134B,* and *HIST1H3H*) were commonly dysregulated among LC, CHRS, and HTCV; *NOX4, CDH13* and *FLJ37035* were commonly dysregulated among LC, T2D, and HTCV. Moreover, LC, AG, HTCV shared three significant genes, namely *ABAT, NDRG2* and *PLCE1*, and *KIF15* and *HIST1H2AI* play a mutual role in LC, SM, CHRIS. *NOX4* was common among LC, OB, T2D and HTCV while that of LC, HTBV, and CHRIS was *NUSAP1*. We observed from Figs. 3 and 4 that CHRS shared the maximum genes with LC.

### Integration of TCGA RNA-seq data and clinical features with patient demographics for prognostic modeling in liver cancer

We obtained mRNA-seq and clinical data for LC patients from the cBioPortal database. The dataset included a total of 442 patient cases with 84 clinical variables, of which 373 cases had corresponding RNA-seq gene expression profiles, covering 20,440 genes. To investigate the prognostic significance of genes associated with LC and its eight identified risk factors, we compared the 230 shared DEGs with the TCGA mRNA-seq data. This comparison yielded 215 genes with available expression values.

From the 84 clinical features, we selected eight key attributes based on their clinical relevance to LC prognosis: Race Category, Diagnosis Age, Sex, Histologic Grade, Cancer Stage, Person Neoplasm Status, Days to Last Follow-up, and Censor Status. These variables were chosen to ensure biological interpretability and to capture essential demographic, diagnostic, and outcome related information.

**Figure 5:**
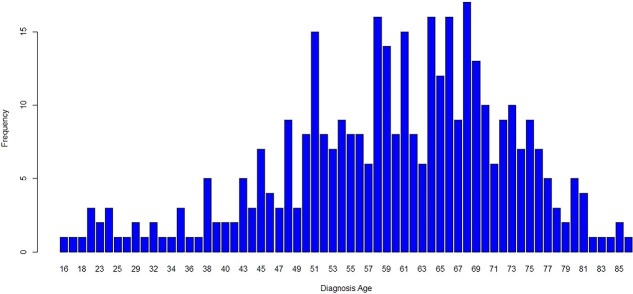
Age Distribution of Patients.

Using this integrated dataset, we applied machine learning–based modeling and Cox proportional hazards regression to assess the relative contributions of both clinical features and gene expression levels to patient survival outcomes in LC. This analysis enabled the identification of key biomarkers and clinical variables with potential prognostic value. To further contextualize our prognostic analysis, we summarized key characteristics of the patient cohort. The age distribution of patients is shown in Fig. 5. The average age of the patients at the time of their diagnoses was 59.47 years, with a range between 16 and 90 years old. The descriptive summary statistics of these factors are shown in Table. From Table 2, it was observed that the LC stage variable has the highest number (10) of categories and 46.56% of all patients are of stage I. There are 5 categories for histology type with the highest percentage of patients (47.88%) being G2, *i.e.* Moderately differentiated, Regarding ethnicity, most patients (49.74%) are from the WHITE population.

**Table 2:** Descriptive Statistics of Clinical Predictors

Characteristics	Category	Frequency	Percentages %
Race	AMERICAN INDIAN OR ALASKA NATIVE	2	0.529101
	ASIAN	161	42.59259
	BLACK OR AFRICAN AMERICAN	17	4.497354
	Others	10	2.645503
	WHITE	188	49.73545
Gender	Female	122	32.27513
	Male	256	67.72487
Cancer stage	Stage I	176	46.56085
	Stage II	87	23.01587
	Stage III	3	0.793651
	Stage IIIA	65	17.19577
	Stage IIIB	9	2.380952
	Stage IIIC	9	2.380952
	Stage IV	2	0.529101
	Stage IVA	1	0.26455
	Stage IVB	2	0.529101
	No Information	24	6.349206
Person Neoplasm Status	No Information	28	7.407407
	Tumor Free	236	62.43386
	With Tumor	114	30.15873
Histologic_grade	G1	55	14.55026
	G2	181	47.8836
	G3	124	32.80423
	G4	13	3.439153
	No Information	5	1.322751

### Survival prediction of significant genes

**Figure 6:**
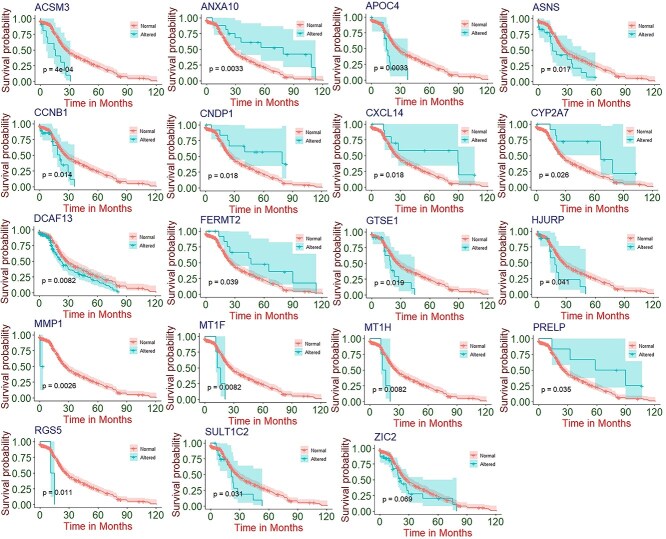
Survival pattern of altered and normal(non-altered) groups for ACSM3, ANXA10, APOC4, ASNS, CCNB1, CNDP1, CXCL14, CYP2A7, DCAF13, FERMT2, GTSE1, HJURP, MMP1, MT1F, MT1H, PRELP, RGS5, SULT1C2, and ZIC2.

To assess the clinical relevance of the identified genes, we performed survival analysis using RNA-seq and clinical data from cBioPortal. We constructed survival curves using the product limit (PL) estimator, comparing altered *versus* non-altered gene expression groups. From this analysis, 19 genes (ASNS, MMP1, RGS5, SULT1C2, DCAF13, ZIC2, GTSE1, HJURP, CCNB1, ACSM3, PRELP, FERMT2, MT1F, CXCL14, CYP2A7, MT1H, CNDP1, ANXA10, APOC4) were identified as significantly associated with patient survival based on p-values <0.05. Kaplan–Meier survival curves comparing altered and normal (non-altered) expression groups for these 19 genes illustrated differences in patient survival outcomes (Fig. 6). While several genes (*e.g.* ASNS, MMP1, RGS5, SULT1C2, DCAF13) exhibited altered expression patterns associated with lower overall survival, others such as ANXA10, CNDP1, CXCL14, CYP2A7, FERMT2 and PRELP were associated with better survival outcomes. These results suggest that the directionality of prognostic influence varies across different genes, underscoring the importance of gene-specific survival associations.

### Cox proportional hazards model for survival risk prediction

We employed the Cox proportional hazards (PH) model to evaluate hazard ratios (HRs) for each gene individually and in combination. This approach allowed us to assess the relative risk of mortality in LC associated with each gene separately, as well as collectively across all genes, enabling the identification of those most strongly linked to patient survival. To perform this analysis, we conducted both univariate (analyzing each gene independently) and multivariate (considering the effects of all other genes) Cox PH regression analyses on a set of 215 genes. Table 3 presents only those genes with statistically significant results (p-value <0.05), including their estimated coefficients (β), hazard ratios (HRs), and p-values. The univariate analysis identified 19 genes— *ASNS*, *MMP1*, *RGS5*, *SULT1C2*, *DCAF13*, *ZIC2*, *GTSE1*, *HJURP*, *CCNB1*, *ACSM3*, *PRELP*, *FERMT2*, *MT1F*, *CXCL14*, *CYP2A7*, *MT1H*, *CNDP1*, *ANXA10*, and *APOC4*—with p-values below 0.05, indicating significant associations with survival in LC patients.

**Table 3:** Significant genes obtained from Univariate and Multivariate Cox Proportional Hazard Model for mRNA Seq data

Genes	Univariate				Genes	Multivariate		
	β	HR	*P*-value	Risk Factors		β	HR	*P*-value
ASNS	6.07E-01	1.84E+00	.02	AG	CDH13	−3.36E+00	3.48E-02	.01
MMP1	2.48E+00	1.19E+01	.02	HTCV	HIST1H3H	1.52E+00	4.55E+00	.01
RGS5	1.63E+00	5.10E+00	.02	SMK	HIST1H2AM	−1.31E+00	2.69E-01	.05
SULT1C2	6.29E-01	1.88E+00	.04	SMK	RGS5	1.32E+01	5.55E+05	.05
DCAF13	3.61E-01	1.44E+00	.01	T2D	SULT1C2	2.00E+00	7.40E+00	.02
ZIC2	6.11E-01	1.84E+00	.01	CHRS,T2D	SUV39H2	2.69E+00	1.47E+01	.01
GTSE1	6.76E-01	1.97E+00	.02	CHRS	ZIC2	1.41E+00	4.08E+00	.03
HJURP	6.76E-01	1.97E+00	.04	CHRS	NEK2	−3.55E+00	2.87E-02	.00
CCNB1	6.68E-01	1.95E+00	.02	CHRS	SIPA1L2	−1.27E+00	2.81E-01	.02
ACSM3	1.09E+00	2.98E+00	.00	CHRS,HTCV	PODXL	3.78E+00	4.37E+01	.01
PRELP	−1.02E+0	3.62E-01	.05	HTCV	CCNB2	3.68E+00	3.97E+01	.00
FERMT2	−6.93E-01	5.00E-01	.04	AG	CD300A	−2.19E+00	1.12E-01	.03
MT1F	1.25E+00	3.48E+00	.01	CHRS	TACSTD2	2.44E+00	1.15E+01	.05
CXCL14	−9.41E-01	3.90E-01	.02	CHRS	SYNPO2	−1.33E+01	1.71E-06	.02
CYP2A7	−8.99E-01	4.07E-01	.03	CHRS	IGFBP5	1.68E+01	1.97E+07	.01
MT1H	1.25E+00	3.48E+00	.01	CHRS	SLC1A2	−1.66E+00	1.90E-01	.03
CNDP1	−9.40E-01	3.91E-01	.02	CHRS	ACSM3	3.15E+00	2.33E+01	.01
ANXA10	−9.28E-01	3.95E-01	.01	CHRS	CD247	3.04E+00	2.10E+01	.03
APOC4	9.20E-01	2.51E+00	.01	ALC	FAM134B	−2.53E+00	7.98E-02	.00
					CAMK2B	2.03E+00	7.58E+00	.02
					NDRG2	3.67E+00	3.91E+01	.01
					FERMT2	−1.80E+00	1.65E-01	.04
					SRD5A1	1.46E+00	4.30E+00	.03
					APOF	2.38E+00	1.08E+01	.00
					CXCL14	−4.28E+00	1.38E-02	.00
					KBTBD11	2.76E+00	1.58E+01	.00
					CYP4A22	−3.66E+00	2.56E-02	.02
					BBOX1	2.70E+00	1.49E+01	.01
					CYP4A11	−2.61E+00	7.33E-02	.04
					VIPR1	−2.87E+00	5.69E-02	.01
					PROZ	−3.35E+00	3.49E-02	.01
					PDLIM5	−2.91E+00	5.45E-02	.05
					APOC4	2.44E+00	1.15E+01	.04

Table 3 summarizes univariate, RF-connected, and multivariate analyses with corresponding hazard ratios (HR) and p-values. We identified 34 significant genes in the multivariate model (see Table 3). We obtained only 7 (RGS5, SULT1C2, CSM3, CXCL14, ZIC2, FERMT2, and POC4) significant genes that are significant in both analyses. In our comparative survival analysis, we identified only seven genes—RGS5, SULT1C2, CSM3, CXCL14, ZIC2, FERMT2, and POC4—that were consistently significant in both the risk-associated and independent dataset analyses. Notably, most of these genes exhibited reduced survival in patients with altered expression, underscoring their potential prognostic relevance. However, among them, FERMT2 showed a distinct expression-survival pattern, and due to its unique behavior, we focused further on the multivariate Cox model, which better accounts for gene–gene interactions and confounding variables, thereby providing a more reliable insight into the prognostic value of these genes.

### Modeling hazard on the combined model containing both clinical and RNAseq data

We have performed multivariate Cox PH regression analysis with both clinical and mRNA-Seq data simultaneously. After removing 26 observations with missing data values, here the total number of events (lives and deaths), n = 346 remained, with 232 events (deaths). Table 4 presents the summary of Cox PH regression results for clinical factors, while Table 5 summarizes the significant genes.

**Table 4:** Summary of the Cox Proportional Hazard Model for combined mRNA seq and Clinical data, showing Clinical data

Variables	β	HR	*P*-value
Diagnosis Age	−1.43E-02	9.86E-01	.4682
Male	8.17E-01	2.26E+00	.1470 3
cancer_stage Stage II	9.27E-01	2.53E+00	.08744
Stage III	−4.43E-01	6.42E-01	.89406
Stage IIIA	5.00E-01	1.65E+00	.5119
Stage IIIB	−8.61E-01	4.23E-01	.62691
**Stage IIIC**	3.99E+00	5.40E+01	**.01386**
**Stage IV**	9.37E+00	1.17E+04	**.01548**
Stage IVA	9.38E+00	1.18E+04	.21448
Stage IVB	−8.25E+00	2.60E-04	.1598
Histologic_Grade G2	7.97E-01	2.22E+00	.3207
G3	−1.15E+00	3.16E-01	.17441
G4	3.96E-01	1.49E+00	.80422
Race ASIAN	6.75E-01	1.96E+00	.8647
BLACK OR AFRICAN AMERICAN	−1.97E+00	1.40E-01	.62972
Others	−1.27E-01	8.81E-01	.97681
WHITE	−6.73E-01	5.10E-01	.85983

Bold/italic values indicate statistically significant clinical parameters.

**Table 5:** Significant genes obtained from the Cox Proportional Hazard Model that used mRNA seq and Clinical data

Genes	Status	β	HR	*P*-value
NUSAP1	Altered	−5.50E+00	4.10E-03	**.023**
CDH13	Altered	−4.13E+00	1.61E-02	**.038**
HIST1H3H	Altered	2.40E+00	1.10E+01	**.020**
HIST1H2AM	Altered	−2.99E+00	5.02E-02	**.002**
HIST1H2AL	Altered	2.53E+00	1.26E+01	**.031**
RGS5	Altered	3.39E+01	5.23E+14	**.011**
SULT1C2	Altered	3.16E+00	2.36E+01	**.016**
SUV39H2	Altered	3.60E+00	3.66E+01	**.025**
CDC25C	Altered	−3.11E+00	4.47E-02	**.049**
SIPA1L2	Altered	−1.88E+00	1.52E-01	**.021**
PODXL	Altered	4.66E+00	1.06E+02	**.016**
IGF2BP3	Altered	−2.73E+00	6.49E-02	**.048**
CDCA5	Altered	−8.19E+00	2.79E-04	**.003**
SEMA6D	Altered	−1.35E+01	1.34E-06	**.005**
CD300A	Altered	−3.36E+00	3.47E-02	**.020**
SYNPO2	Altered	−2.97E+01	1.28E-13	**.006**
IGFBP5	Altered	3.98E+01	1.99E+17	**.000**
SOCS2	Altered	3.92E+00	5.05E+01	**.003**
FOXP2	Altered	4.73E+00	1.14E+02	**.031**
TDO2	Altered	4.00E+00	5.44E+01	**.006**
ALDH6A1	Altered	3.56E+00	3.52E+01	**.015**
ACSM3	Altered	5.47E+00	2.39E+02	**.014**
TMEM47	Altered	5.12E+00	1.67E+02	**.023**
METTL7A	Altered	−5.50E+00	4.10E-03	**.028**
CD247	Altered	1.09E+01	5.16E+04	**.000**
FAM134B	Altered	−4.30E+00	1.36E-02	**.000**
ABCA1	Altered	−3.46E+00	3.14E-02	**.023**
GATM	Altered	2.65E+00	1.42E+01	**.046**
PTPRD	Altered	−6.11E+00	2.22E-03	**.008**
PFKFB1	Altered	3.44E+00	3.12E+01	**.030**
DSE	Altered	3.99E+00	5.39E+01	**.015**
SRD5A1	Altered	2.09E+00	8.11E+00	**.024**
DBH	Altered	4.15E+01	1.05E+18	**.000**
TMEM27	Altered	−4.83E+00	8.00E-03	**.017**
CYP2C8	Altered	−4.22E+00	1.47E-02	**.014**
CFP	Altered	1.22E+01	2.03E+05	**.031**
MARCO	Altered	−2.55E+01	8.14E-12	**.001**
FAM65C	Altered	−7.09E+00	8.37E-04	**.022**
LYVE1	Altered	9.49E+00	1.33E+04	**.000**
APOF	Altered	2.79E+00	1.64E+01	**.008**
ATOH8	Altered	3.01E+00	2.03E+01	**.014**
CXCL14	Altered	−6.14E+00	2.15E-03	**.001**
IGFALS	Altered	−7.93E+00	3.59E-04	**.002**
PTH1R	Altered	6.98E+00	1.08E+03	**.000**
CLEC4G	Altered	−7.48E+01	3.38E-33	**.000**
SLC27A5	Altered	−4.12E+00	1.62E-02	**.020**
GLYAT	Altered	5.62E+00	2.77E+02	**.008**
DNASE1L3	Altered	4.40E+00	8.11E+01	**.010**
VIPR1	Altered	−5.13E+00	5.89E-03	**.006**
FXYD1	Altered	2.18E+00	8.82E+00	**.032**
ECM1	Altered	−1.37E+01	1.09E-06	**.000**
ZFP1	Altered	−5.67E+00	3.44E-03	**.005**
LRRN3	Altered	−1.11E+01	1.48E-05	**.015**
TMEM56	Altered	5.83E+00	3.41E+02	**.014**

Bold/italic values indicate statistically significant mRNA expression.

For clinical factors, we observed that patients with Stage IIIC of LC are 54 times less likely to survive compared to stage I of LC, and patients with Stage IV had an approximately 11,700-fold increased risk of death compared to Stage I.

For gene expression data, hazard analysis was conducted using 215 significant genes, of which 54 showed a significant association with patient survival (Table 5).

**Figure 7:**
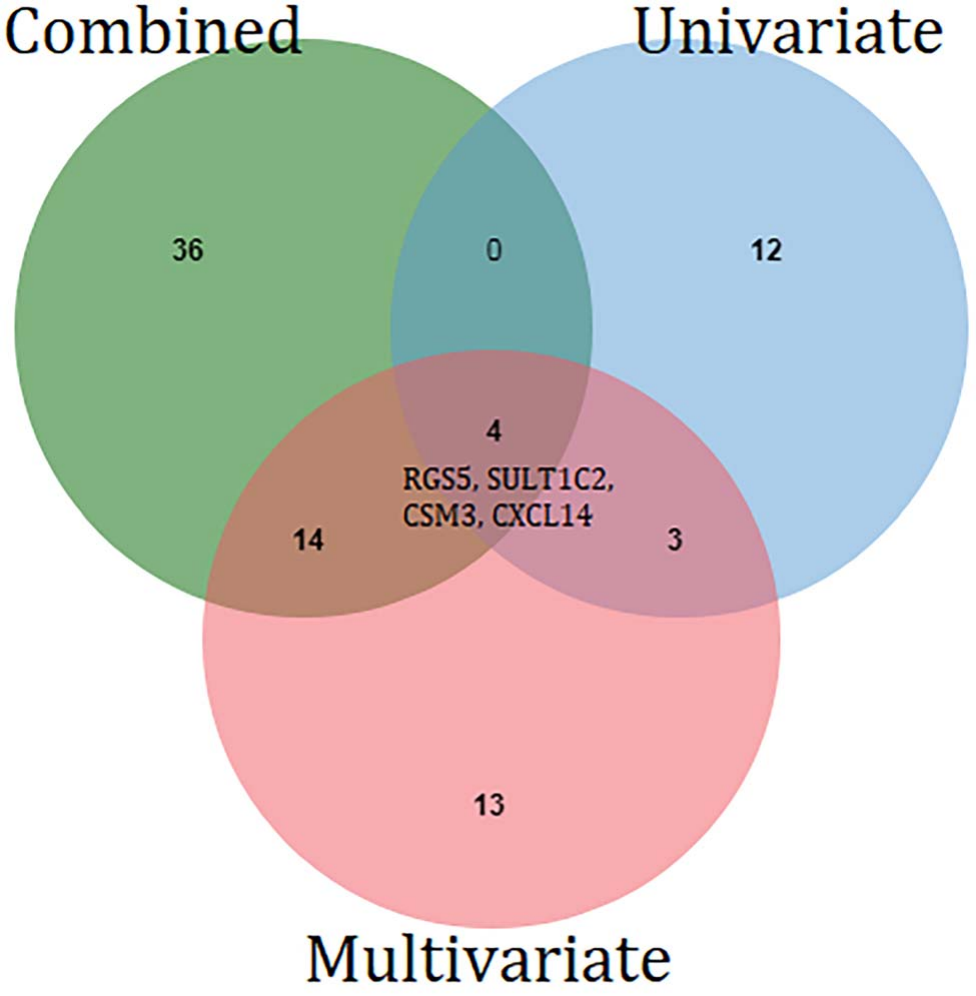
Venn diagram of significant genes found in univariate, multivariate and combined hazard model analysis.

A Venn diagram of the significant genes summarizes the relationships between altered gene expression and risk of death identified using different methodologies and is shown in Fig. 7. We identified 18 common genes analysis (CDH13, HIST1H3H, HIST1H2AM, RGS5, SULT1C2, SUV39H2, SIPA1L2, PODXL,CD300A, SYNPO2, IGFBP5, ACSM3, CD247, FAM134B, SRD5A1, APOF, CXCL14, VIPR1) between combined analysis and multivariate analysis. Importantly, among the 18 common genes, only four (RGS5, SULT1C2, ACSM3, and CXCL14) consistently emerged as survival-associated markers across the multivariate and combined Cox PH models, underscoring their robustness as potential prognostic biomarkers. From multivariate analysis and Cox PH regression analysis on combined data, we found that 70 genes among the 215 studied showed a significant association with risk of death. Gene expression of ZIC2, FERMT2 and POC4 were significant in both univariate and multivariate models, that of 14 genes common in multivariate and combined hazard models.

### Comparison between risk-associated and independent survival analyses

After filtering, 967 genes from the independent transcriptomic dataset were retained for survival analysis. Univariate Cox regression identified 76 genes significantly associated with overall survival, while multivariate and combined Cox models revealed 376 and 335 significant genes, respectively. In parallel, survival analyses on the 215 risk-associated genes identified 19 genes through univariate, 34 through multivariate, and 54 through combined models. Notably, 18 of the 19 univariate genes from the risk-associated set overlapped with those identified in the independent univariate analysis, with ZIC2 being uniquely significant only in the risk-associated set. Additionally, we observed 17 and 25 overlapping genes in the multivariate and combined analyses, respectively.

This comparison demonstrates that a substantial portion of risk-associated genes are also relevant to survival prediction, thereby reinforcing their potential clinical importance, while also revealing additional survival markers that were missed when restricting analysis to only risk associated genes.

### Enrichment analysis

**Figure 8:**
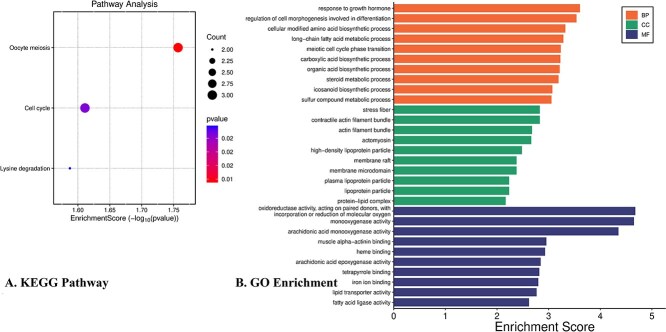
Gene ontology terms and Pathway associated with the selected 70 significantly associated genes with the Liver Cancer.

To uncover the biological relevance of the identified genes, we performed enrichment analysis on the 70 significant genes derived from multivariate, and combined Cox PH models. Using clusterProfiler R package, we identified key pathways and GO enrichment processes associated with liver cancer. We observed that three significant pathways (see Fig. 8A) are associated with the significantly regulated genes of Liver Cancer. We further conducted Gene Ontology (GO) enrichment analysis of the identified significant genes using the clusterProfiler R package. As illustrated in Fig. 8B, the analysis revealed the top 10 enriched terms across Biological Processes (BP), Cellular Components (CC), and Molecular Functions (MF), providing insights into the functional roles of these genes.

### Proteomics signatures: hub target proteins from PPI analysis

**Figure 9:**
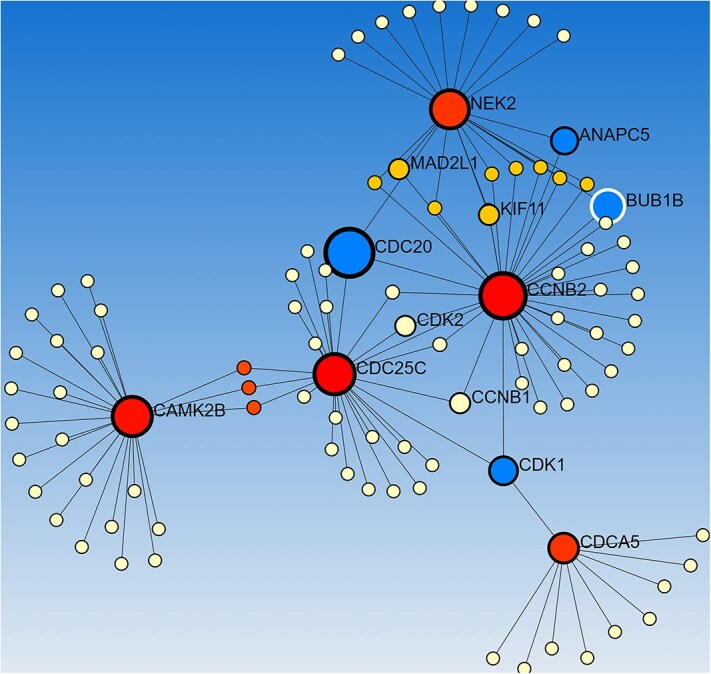
Exploring the Protein–Protein Interaction Network of 70 Common Genes in LC and Associated Risk Factors.

We constructed PPI network considering the identified 70 unique genes (Fig. 9) and identified ten hub genes namely CCNB2, CDC25C, NEK2,CDCA5, CDK1, PPP1CA, PPP1CC, PPP1CB, CAMK2B, and CDC20 using the topological analysis.

### Validation of protein expression in the HPA database

**Figure 10:**
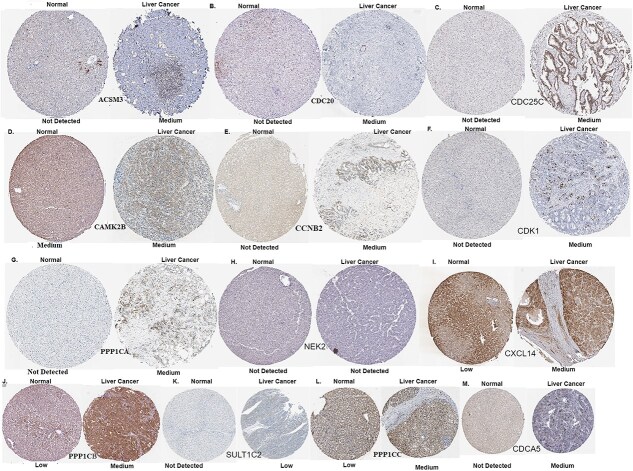
The HPA database provided immunohistochemistry images of hub genes in normal lung tissues and liver cancer tissues. These images displayed protein representations of ACSM3, CDC20, CDC25C, CAMK2B, CCNB2, CDK1, PPP1CA, NEK2, CXCL14, PPP1CB, SULT1C2, PPP1CC, and CDCA5 (identified as A-M).

To further validate our findings, we examined protein expression levels of significant genes using the HPA database. The HPA database was used to explore the protein expression levels of significant biomarkers (hub genes and common genes in three analyses), which are genes that have a high degree of connectivity within a network of genes (see Fig. 10). Notably, CAMK2B showed medium expression in both normal and liver cancer tissues, while CDC20 and PPP1CA were undetected in normal tissue but exhibited medium expression in liver cancer. Similarly, PPP1CB and PPP1CC displayed low expression in normal tissue and medium levels in cancerous tissue. In contrast, genes such as CCNB2, CDC25C, NEK2, CDCA5, CSM3, CDK1, and SULT1C2 were not detected in normal liver but showed medium expression in liver cancer, suggesting cancer-specific up-regulation. CXCL14 had low expression in normal tissue and medium expression in cancer, indicating potential involvement in tumor progression.

## Discussion

Our study aims to uncover significant genes and pathways influencing LC development due to its RFDs by integrating Bioinformatics and Machine Learning approaches. This study addresses a significant knowledge gap, enhancing understanding of LC’s molecular mechanisms and genetic associations with RFDs, with potential implications for disease prediction and therapeutic advancements. Initially, we identified common differentially expressed genes (CDEGs) across three datasets related to LC. Subsequently, we constructed Gene-Disease association networks for eight risk factors to assess their relations with LC. Next, we utilized mRNA and clinical data of LC from TCGA specifically focusing on mRNA data associated with the identified CDEGs. Subsequently, we performed univariate, multivariate, and combined analyses to identify genes and clinical factors that influence the survival of patients with LC. Importantly, the combined dataset of genes and clinical factors was designated as the primary framework for assessing LC survival, with multivariate Cox regression applied solely to refine and validate these findings rather than serve as an equivalent analysis. Finally, PPI network, common pathway, and GO ontology analyses were performed on genes from two analyses (combined and multivariate) to identify hub proteins and significant pathways implicated in LC’s comorbidity with RFDs. This study addresses a significant knowledge gap, enhancing understanding of LC’s molecular mechanisms and genetic associations with RFDs.

Our analyses confirm a strong association between LC and certain risk factors, particularly CHRS (126 genes), HTCV (28), T2D (31 genes), and SMK (15), evidenced by their significant overlap in DEGs. In contrast, HTBV shared fewer DEGs (5 genes). We identified 230 unique genes common to LC and its risk factors through diseasome networks. Subsequently, RNA-seq data from TCGA were collected for these genes, with data available for 215 genes. In addition, six clinical features of TCGA were considered for our analysis.

One of the key novelties of our work is the identification of common genetic markers across multiple RFDs, which could serve as potential therapeutic targets or diagnostic biomarkers. While prior studies have examined LC in isolation or focused on specific risk factors, our approach high lights the shared molecular landscape of LC and its comorbid conditions. For instance, ACSM3, ZIC2, and APOC4 emerged as significant survival-associated genes, offering new avenues for precision medicine in LC. In the subsequent phase, univariate analysis was conducted on the 215 genes, revealing 19 significant genes. To determine the most influential genes, both univariate (per gene) and multivariate (considering all other genes) Cox proportional hazards models were applied to each gene. Among these, ACSM3 exhibited the lowest p-value, suggesting a significant association with LC patient survival. ACSM3, linked to SMK and liver CHRS, has been identified as a potential LC biomarker by Ruan *et. al* (2017) [30]. Additionally, APOC4, associated with ALC, and ZIC2, associated with CHRS and T2D, were found to influence LC survival. ASNS was associated with AG. Overall, LC survival is influenced by SMK, ALC, T2D, HTCV, AG, and CHRS.

In the combined Cox PH model integrating clinical and RNA-Seq data, advanced disease stages (IIIC and IV) and 54 genes were significantly associated with overall survival (Tables 4 and 5). This model, which incorporates both molecular and clinical variables, provides a more comprehensive framework for identifying prognostic markers in liver cancer. Cross-comparison with the multivariate Cox regression model (genes only) revealed 18 common genes between the two approaches. Among these, four genes (RGS5, SULT1C2, ACSM3, and CXCL14) consistently emerged as survival-associated markers, underscoring their robustness and reliability as potential prognostic biomarkers. Previous studies further support their clinical relevance: RGS5 has been implicated in liver cancer recurrence, venous infiltration, and poor survival [31]; SULT1C2 has been strongly associated with LC [32]; CXCL14 is recognized as a tumor suppressor and prognostic factor [33]; and ACSM3 has also been reported in LC progression [34].

In the multivariate gene-only analysis, 34 genes were found significant (Table 3), of which seven—including RGS5, CXCL14, SULT1C2, APOC4, ZIC2, ACSM3 and FERMT2—also showed significance in univariate testing. While most were associated with worse outcomes [31–35], FERMT2 was uniquely linked to improved survival. As multivariate models adjust for joint effects, these findings enhance the reliability of the overlapping candidates while also pointing to additional genes of potential interest.

To contextualize these findings at the systems level, a PPI network was constructed from all 70 significant genes identified across the analyses. This revealed ten hub genes (CCNB2, CDC25C, NEK2, CDCA5, CDK1, PPP1CA, PPP1CC, PPP1CB, CAMK2B, and CDC20), many of which are established drivers of LC progression, cell-cycle dysregulation, and poor prognosis. Their emergence underscores the biological plausibility of our integrative framework and strengthens the potential for clinical translation.

Li *et al.* suggest that CCNB2 may serve as a prognostic factor and play a role in LC genesis and progression [36]. Thong *et al.* identified CCNB1, NEK2, and CDK1 as prognostic biomarkers for liver cancer [37]. CDCA5 is proposed as a potential LC biomarker by Bowel *et al.* [38]. CDC20, a key regulator in the cell cycle, is overexpressed in HCC and correlates with tumor characteristics and poor prognosis [39, 40]. Bioinformatic analyses have revealed other important genes in HCC, including BIRC5 and UBE2C, which are linked to disease progression and survival [40]. In hepatoblastoma, CCNA2, CDK1, and CDC20 were identified as potential therapeutic targets, with their knockdown inhibiting aggressive cell behaviors [41]. A genetic variant of PPP1CB (rs13025377) was found to influence the risk of hepatitis B virus-related hepatocellular carcinoma (HCC) in Han Chinese populations [42]. From our KEGG pathway analysis, genes including CCNB2, and CDC25C are associated with Oocyte meiosis Lysine degradation and cell cycle regulation. Utilizing 70 significant genes, we identified 3 significant KEGG path ways (see Fig. 8A). Independent literature review confirms robust connections between the Cell cycle, and oocyte meiosis pathways with LC [43, 44]. Another noteworthy pathway is *lysine degradation*, which plays a pivotal role in liver cancer progression by modulating key cellular processes such as metabolic regulation, protein post-translational modifications, and gene expression [45–47]. Alterations in lysine-derived modifications—such as acetylation and lactylation—have been shown to influence cancer cell proliferation, migration, and tumor development, thereby highlighting their potential as targets for therapeutic intervention [45–47].

A notable limitation of this study is the absence of external validation cohorts and experimental methods, such as qPCR or immunohistochemistry, which are essential for further validating the identified biomarkers. By integrating transcriptomic and clinical data, our study not only confirms previously known associations but also uncovers novel genetic links between LC and its risk factors. These findings contribute to a deeper understanding of LC’s molecular mechanisms and may help refine risk assessment, biomarker discovery, and potential therapeutic targets.

## Conclusions

Our integrative computational framework identified robust biomarkers and clinical factors with strong diagnostic and prognostic value in liver cancer (LC). By applying a stepwise strategy—combined analysis followed by multivariate Cox regression—we prioritized overlapping significant genes as reliable prognostic markers, with univariate results used only for comparison. Four survival-associated genes (RGS5, SULT1C2, ACSM3, CXCL14) and ten hub genes (CCNB2, CDC25C, NEK2, CDCA5, CDK1, PPP1CA, PPP1CC, PPP1CB, CAMK2B, CDC20) consistently emerged as key drivers of LC progression. Clinically, advanced-stage (IIIC/IV) patients had markedly poorer survival, with demographic data pointing to higher susceptibility among white males aged 60–70.

Network-based analyses further revealed strong molecular associations between LC and its major risk factors, with cirrhosis demonstrating the highest gene overlap, followed by hepatitis C and type 2 diabetes. These findings underscore cirrhosis as the most critical comorbidity contributing to LC risk. Collectively, our results highlight clinically actionable biomarkers and pathways that underscore the importance of experimental validation and hold promise for guiding precision diagnostics and targeted therapies in LC.

**Key points**

- Applies an integrative bioinformatics and machine learning framework to systematically dissect liver cancer (LC) biology and elucidate its molecular interplay with major risk factors.
- Derives shared molecular signatures by cross-analyzing transcriptomic datasets from LC and associated risk conditions, enabling construction of gene–disease interaction networks.
- Identifies robust prognostic biomarkers through Cox proportional hazards modeling of integrated RNA-seq and clinical data, highlighting genes linked to patient survival.
- Highlights four consistently survival-associated genes (RGS5, SULT1C2, ACSM3, CXCL14) and uncovers ten key regulatory hub genes through protein–protein interaction analysis and functional enrichment profiling.
- Establishes a transferable analytical framework applicable to diverse cancer types for systematic identification of prognostic genes, pathways, and clinically relevant factors.

## Supplementary Material

Supplementary_File-1_elaf019

Supplementary_File-2_elaf019

Supplementary_File-3_elaf019

Revised-Manuscripts_R2-BFGP-24-0136_elaf019

## Data Availability

We retrieved anonymized clinical data and RNA-seq expression profiles for Liver Hepatocellular Carcinoma (LC; TCGA, Provisional) from the cBioPortal for Cancer Genomics (https://www.cbioportal.org/). Additionally, transcriptomic datasets used in this study were obtained from the NCBI GEO database. The accession numbers of the ten datasets analyzed are as follows: GSE134921, GSE101728, and GSE101685 for liver cancer (LC); GSE102451 for CHRS; GSE110312 for hepatitis C virus (HTCV); GSE135501 for HTBV; GSE674 for aging (AG); GSE52553 for alcoholism (ALC); GSE23343 for T2D; GSE48964 for OB; and GSE4806 for SMK. All datasets are publicly available and can be accessed through the NCBI GEO repository (https://www.ncbi.nlm.nih.gov/geo/) and cBioPortal.

## Notes

Tania Akter Asa, a PhD student at Jagannath University, focuses on bioinformatics, machine learning, and data science, with experience in teaching and biomedical data-driven research.

Dr. Md Ali Hossain is an Associate Professor at DIU, Director, NanoBio Technology Center, DIU and completed his PhD from Jahangirnagar University, he has 14 years of teaching and 9 years of research experience in bioinformatics, machine learning, and data science.

Dr. Md. Shahjahan Ali is a Professor of EEE at Islamic University, specializing in wireless networking, wireless communication, and image processing, with 25 years of teaching and research experience.

Dr. Md. Zulfiker Mahmud is a Professor in the Department of Computer Science and Engineering at Jagannath University, specializing in IoT, Machine Learning, and intelligent systems.

Dr. Moni, with a PhD from Cambridge and postdoctoral training at UNSW and Sydney, specializes in AI, bioinformatics, and health data science, advancing interpretable models for disease diagnosis and healthcare.

Prof. Dr. Mohammad Zahidul Rahman is a renowned academic in Computer Science and Engineering, with expertise in E-Commerce, Computer Security, E-Governance, Communication, Machine Learning, and Bioinformatics.
