# Supplementary material for: Unraveling risk factors and transcriptomic signatures in liver cancer progression and mortality through machine learning and bioinformatics
Source: Brief Funct Genomics. 2026 Jan 9;25:elaf019. doi: 10.1093/bfgp/elaf019 (PMC12785888; doi:10.1093/bfgp/elaf019)
Supplement: Revised-Manuscripts_R2-BFGP-24-0136_elaf019 [file revised-manuscripts_r2-bfgp-24-0136_elaf019.zip › Ali_LC_BIB (1)/Clinical.pdf]

Variables	β	HR	P-Value
AMERICAN INDIAN OR ALASKA NATIVE	2.78E+00	1.61E+01	**0.00074**
ASIAN	-2.73E-02	9.73E-01	0.95317
BLACK OR AFRICAN AMERICAN	7.37E-01	2.09E+00	**0.01637**
NATIVE HAWAIIAN OR OTHER PACIFIC ISLANDER	1.54E+00	4.68E+00	0.16029
age	2.60E-02	1.03E+00	3.30E-05
anatomic_site Peritoneum ovary	3.83E-02	1.04E+00	0.97248
histologic_grade G2	6.95E-01	2.00E+00	0.40454
G3	8.01E-01	2.23E+00	0.33189
G4	2.65E+00	1.42E+01	0.08085
GB	3.36E-01	1.40E+00	0.83559
GX	-1.91E-01	8.26E-01	0.84474
tumour_site Left	-3.71E-01	6.90E-01	**0.05113**
tumour_site Right	2.69E-01	1.31E+00	0.19474
Stage IB	1.22E+00	3.40E+00	0.25561
Stage IC	-7.38E-01	4.78E-01	0.49448
Stage IIA	-1.58E+01	1.43E-07	0.99185
Stage IIB	6.35E-02	1.07E+00	0.93521
Stage IIC	-1.18E+00	3.09E-01	**0.00483**
Stage IIIA	-9.63E-02	9.08E-01	0.90753
Stage IIIB	-2.79E-01	7.57E-01	0.42995
Stage IIIC	-3.10E-01	7.33E-01	0.08836
Stage IV	NA	NA	NA
