# Supplementary material for: Unraveling risk factors and transcriptomic signatures in liver cancer progression and mortality through machine learning and bioinformatics
Source: Brief Funct Genomics. 2026 Jan 9;25:elaf019. doi: 10.1093/bfgp/elaf019 (PMC12785888; doi:10.1093/bfgp/elaf019)
Supplement: Revised-Manuscripts_R2-BFGP-24-0136_elaf019 [file revised-manuscripts_r2-bfgp-24-0136_elaf019.zip › Ali_LC_BIB (1)/ClinicalDescrip.pdf]

Characteristics	Category	Frequency	Percentages %
Race	AMERICAN INDIAN OR ALASKA NATIVE	2	0.529101
	ASIAN	161	42.59259
	BLACK OR AFRICAN AMERICAN	17	4.497354
	Others	10	2.645503
	WHITE	188	49.73545
Gender	Female	122	32.27513
	Male	256	67.72487
Cancer stage	Stage I	176	46.56085
	Stage II	87	23.01587
	Stage III	3	0.793651
	Stage IIIA	65	17.19577
	Stage IIIB	9	2.380952
	Stage IIIC	9	2.380952
	Stage IV	2	0.529101
	Stage IVA	1	0.26455
	Stage IVB	2	0.529101
	No Information	24	6.349206
Person Neoplasm Status	No Information	28	7.407407
	Turmor Free	236	62.43386
	With Turmor	114	30.15873
Histologic_grade	G1	55	14.55026
	G2	181	47.8836
	G3	124	32.80423
	G4	13	3.439153
	No Information	5	1.322751
