# Supplementary material for: Unraveling risk factors and transcriptomic signatures in liver cancer progression and mortality through machine learning and bioinformatics
Source: Brief Funct Genomics. 2026 Jan 9;25:elaf019. doi: 10.1093/bfgp/elaf019 (PMC12785888; doi:10.1093/bfgp/elaf019)
Supplement: Revised-Manuscripts_R2-BFGP-24-0136_elaf019 [file revised-manuscripts_r2-bfgp-24-0136_elaf019.zip › Ali_LC_BIB (1)/CombinedSig.pdf]

Genes	Status	β	HR	P-Value
NUSAP1	Altered	-5.50E+00	4.10E-03	**0.023**
CDH13	Altered	-4.13E+00	1.61E-02	**0.038**
HIST1H3H	Altered	2.40E+00	1.10E+01	**0.020**
HIST1H2AM	Altered	-2.99E+00	5.02E-02	**0.002**
HIST1H2AL	Altered	2.53E+00	1.26E+01	**0.031**
RGS5	Altered	3.39E+01	5.23E+14	**0.011**
SULT1C2	Altered	3.16E+00	2.36E+01	**0.016**
SUV39H2	Altered	3.60E+00	3.66E+01	**0.025**
CDC25C	Altered	-3.11E+00	4.47E-02	**0.049**
SIPA1L2	Altered	-1.88E+00	1.52E-01	**0.021**
PODXL	Altered	4.66E+00	1.06E+02	**0.016**
IGF2BP3	Altered	-2.73E+00	6.49E-02	**0.048**
CDCA5	Altered	-8.19E+00	2.79E-04	**0.003**
SEMA6D	Altered	-1.35E+01	1.34E-06	**0.005**
CD300A	Altered	-3.36E+00	3.47E-02	**0.020**
SYNPO2	Altered	-2.97E+01	1.28E-13	**0.006**
IGFBP5	Altered	3.98E+01	1.99E+17	**0.000**
SOCS2	Altered	3.92E+00	5.05E+01	**0.003**
FOXP2	Altered	4.73E+00	1.14E+02	**0.031**
TDO2	Altered	4.00E+00	5.44E+01	**0.006**
ALDH6A1	Altered	3.56E+00	3.52E+01	**0.015**
ACSM3	Altered	5.47E+00	2.39E+02	**0.014**
TMEM47	Altered	5.12E+00	1.67E+02	**0.023**
METTL7A	Altered	-5.50E+00	4.10E-03	**0.028**
CD247	Altered	1.09E+01	5.16E+04	**0.000**
FAM134B	Altered	-4.30E+00	1.36E-02	**0.000**
ABCA1	Altered	-3.46E+00	3.14E-02	**0.023**
GATM	Altered	2.65E+00	1.42E+01	**0.046**
PTPRD	Altered	-6.11E+00	2.22E-03	**0.008**
PFKFB1	Altered	3.44E+00	3.12E+01	**0.030**
DSE	Altered	3.99E+00	5.39E+01	**0.015**
SRD5A1	Altered	2.09E+00	8.11E+00	**0.024**
DBH	Altered	4.15E+01	1.05E+18	**0.000**
TMEM27	Altered	-4.83E+00	8.00E-03	**0.017**
CYP2C8	Altered	-4.22E+00	1.47E-02	**0.014**
CFP	Altered	1.22E+01	2.03E+05	**0.031**
MARCO	Altered	-2.55E+01	8.14E-12	**0.001**
FAM65C	Altered	-7.09E+00	8.37E-04	**0.022**
LYVE1	Altered	9.49E+00	1.33E+04	**0.000**
APOF	Altered	2.79E+00	1.64E+01	**0.008**
ATOH8	Altered	3.01E+00	2.03E+01	**0.014**
CXCL14	Altered	-6.14E+00	2.15E-03	**0.001**
IGFALS	Altered	-7.93E+00	3.59E-04	**0.002**
PTH1R	Altered	6.98E+00	1.08E+03	**0.000**
CLEC4G	Altered	-7.48E+01	3.38E-33	**0.000**
SLC27A5	Altered	-4.12E+00	1.62E-02	**0.020**
GLYAT	Altered	5.62E+00	2.77E+02	**0.008**
DNASE1L3	Altered	4.40E+00	8.11E+01	**0.010**
VIPR1	Altered	-5.13E+00	5.89E-03	**0.006**
FXYD1	Altered	2.18E+00	8.82E+00	**0.032**
ECM1	Altered	-1.37E+01	1.09E-06	**0.000**
ZFP1	Altered	-5.67E+00	3.44E-03	**0.005**
LRRN3	Altered	-1.11E+01	1.48E-05	**0.015**
TMEM56	Altered	5.83E+00	3.41E+02	**0.014**
