# Supplementary figures and images for: Unraveling risk factors and transcriptomic signatures in liver cancer progression and mortality through machine learning and bioinformatics
Source: Brief Funct Genomics. 2026 Jan 9;25:elaf019. doi: 10.1093/bfgp/elaf019 (PMC12785888; doi:10.1093/bfgp/elaf019)

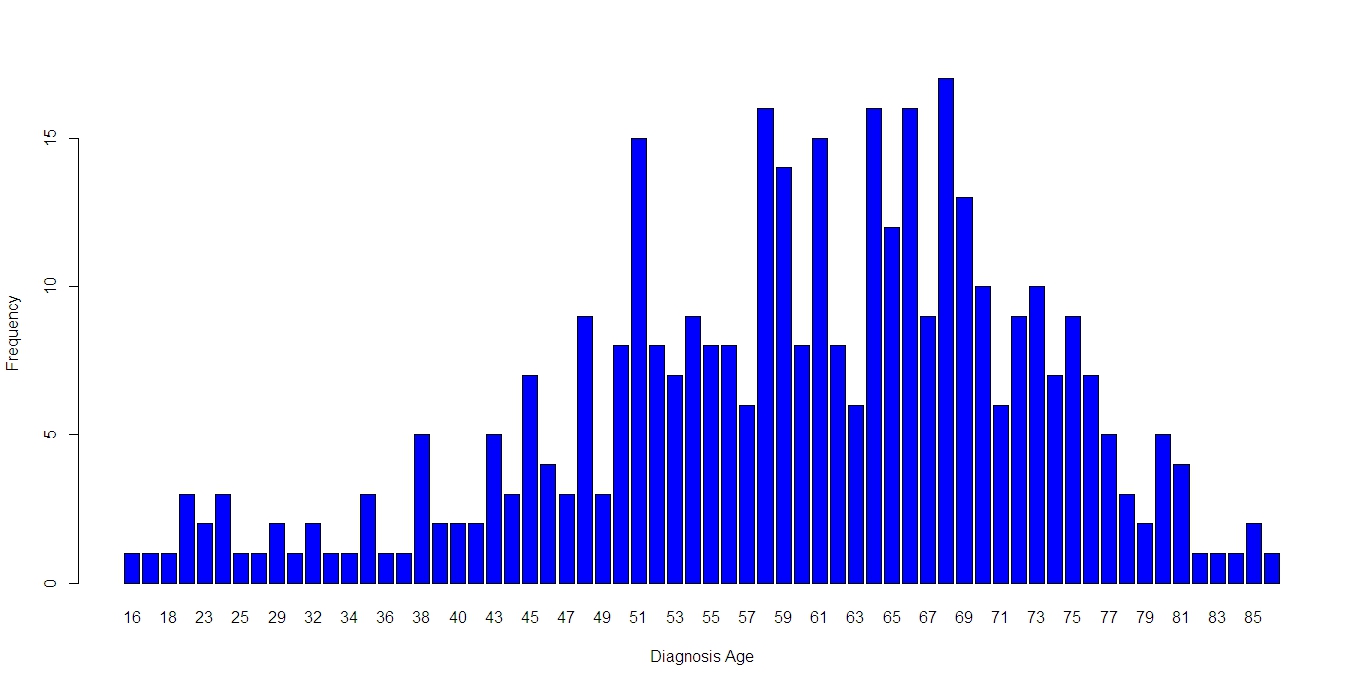

Supplement: Revised-Manuscripts_R2-BFGP-24-0136_elaf019 [file revised-manuscripts_r2-bfgp-24-0136_elaf019.zip › Ali_LC_BIB (1)/AgeDistribution.jpeg]

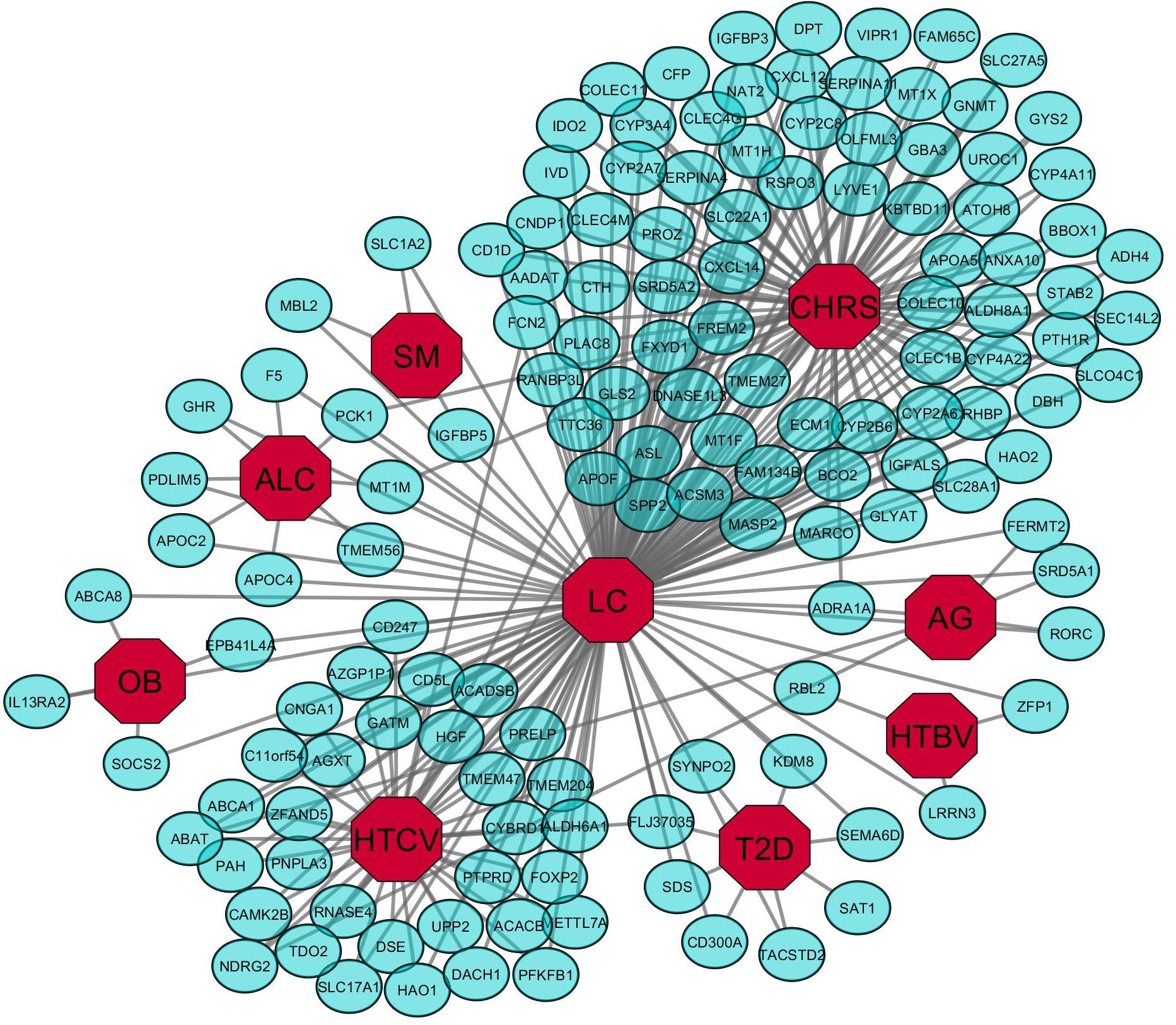

Supplement: Revised-Manuscripts_R2-BFGP-24-0136_elaf019 [file revised-manuscripts_r2-bfgp-24-0136_elaf019.zip › Ali_LC_BIB (1)/DownNetwork.jpeg]

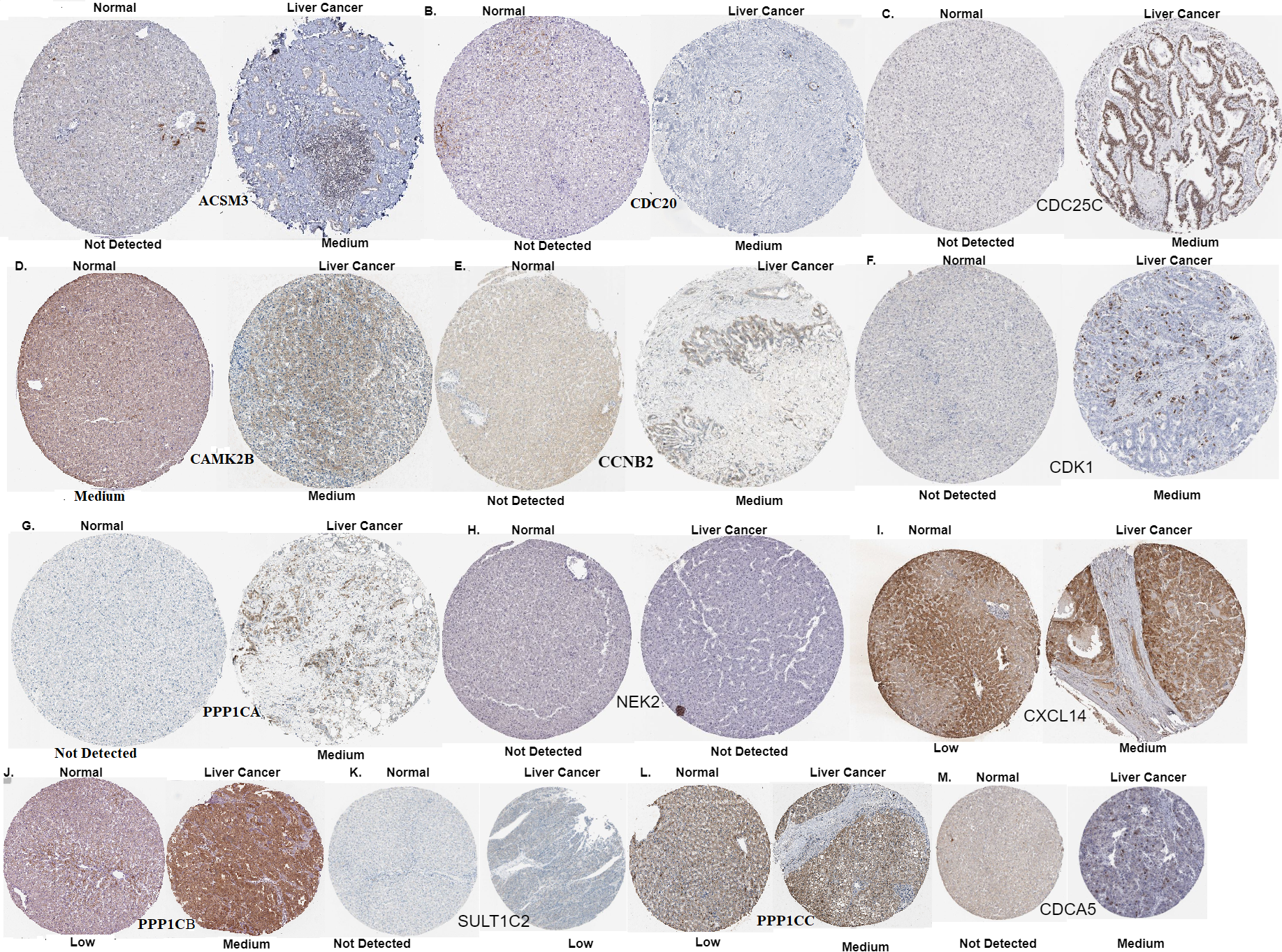

Supplement: Revised-Manuscripts_R2-BFGP-24-0136_elaf019 [file revised-manuscripts_r2-bfgp-24-0136_elaf019.zip › Ali_LC_BIB (1)/HPA1.png]

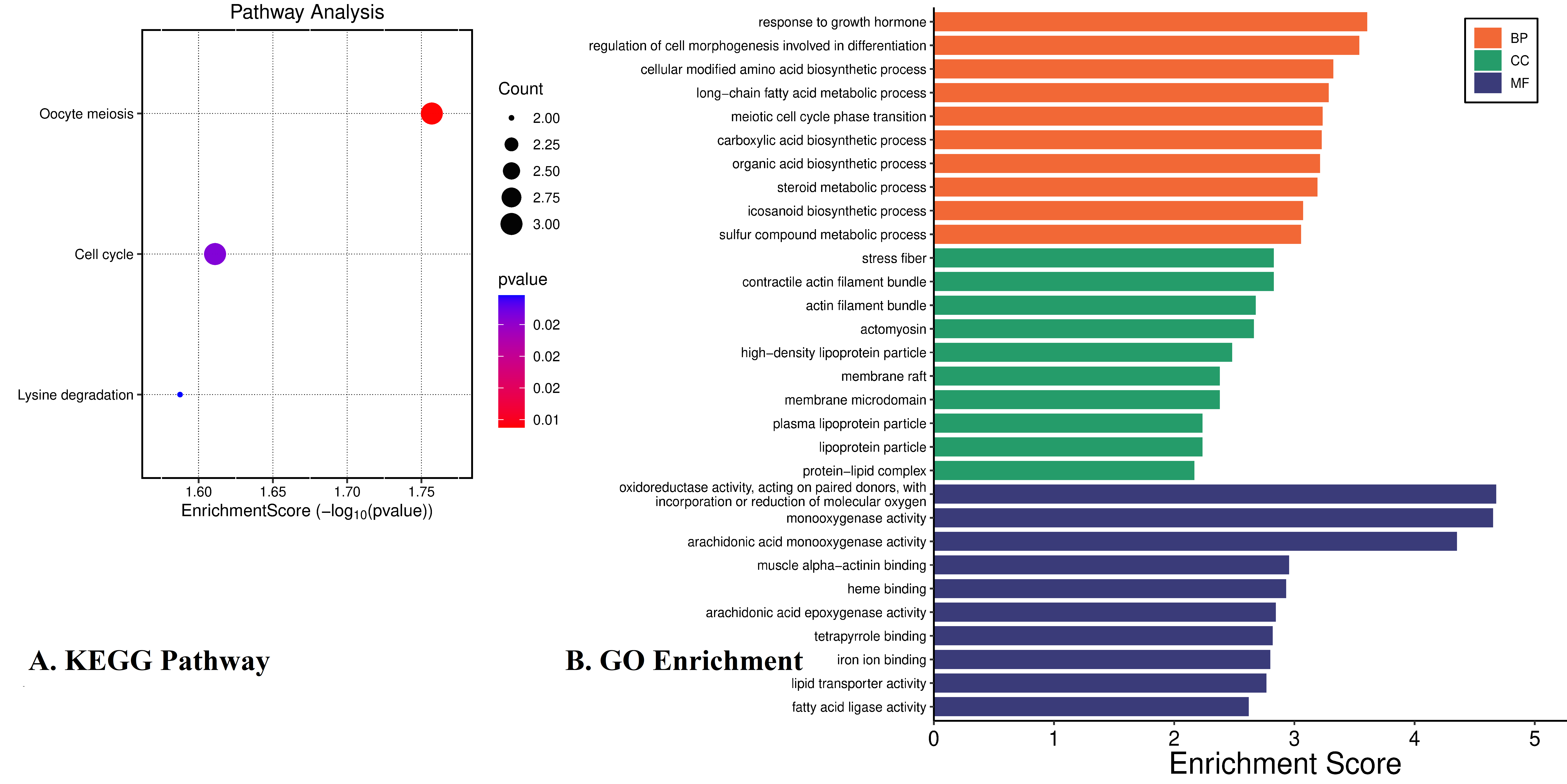

Supplement: Revised-Manuscripts_R2-BFGP-24-0136_elaf019 [file revised-manuscripts_r2-bfgp-24-0136_elaf019.zip › Ali_LC_BIB (1)/kegg-go.png]

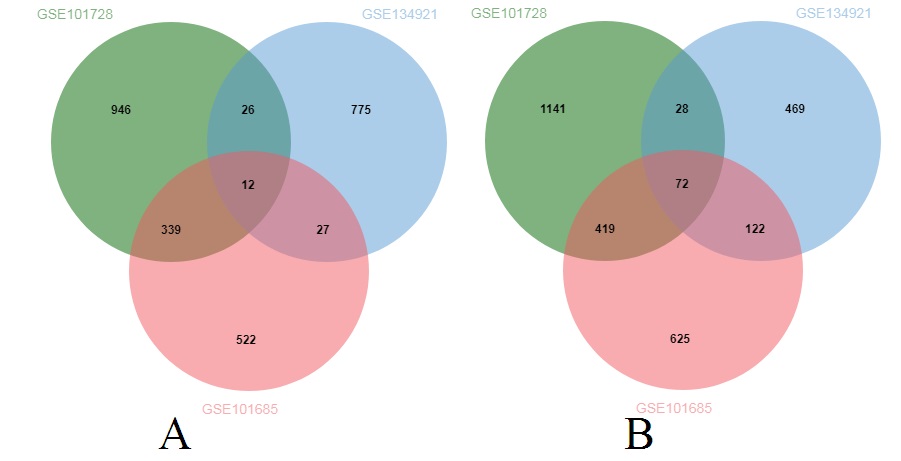

Supplement: Revised-Manuscripts_R2-BFGP-24-0136_elaf019 [file revised-manuscripts_r2-bfgp-24-0136_elaf019.zip › Ali_LC_BIB (1)/LC_Common_Up_Down.jpg]

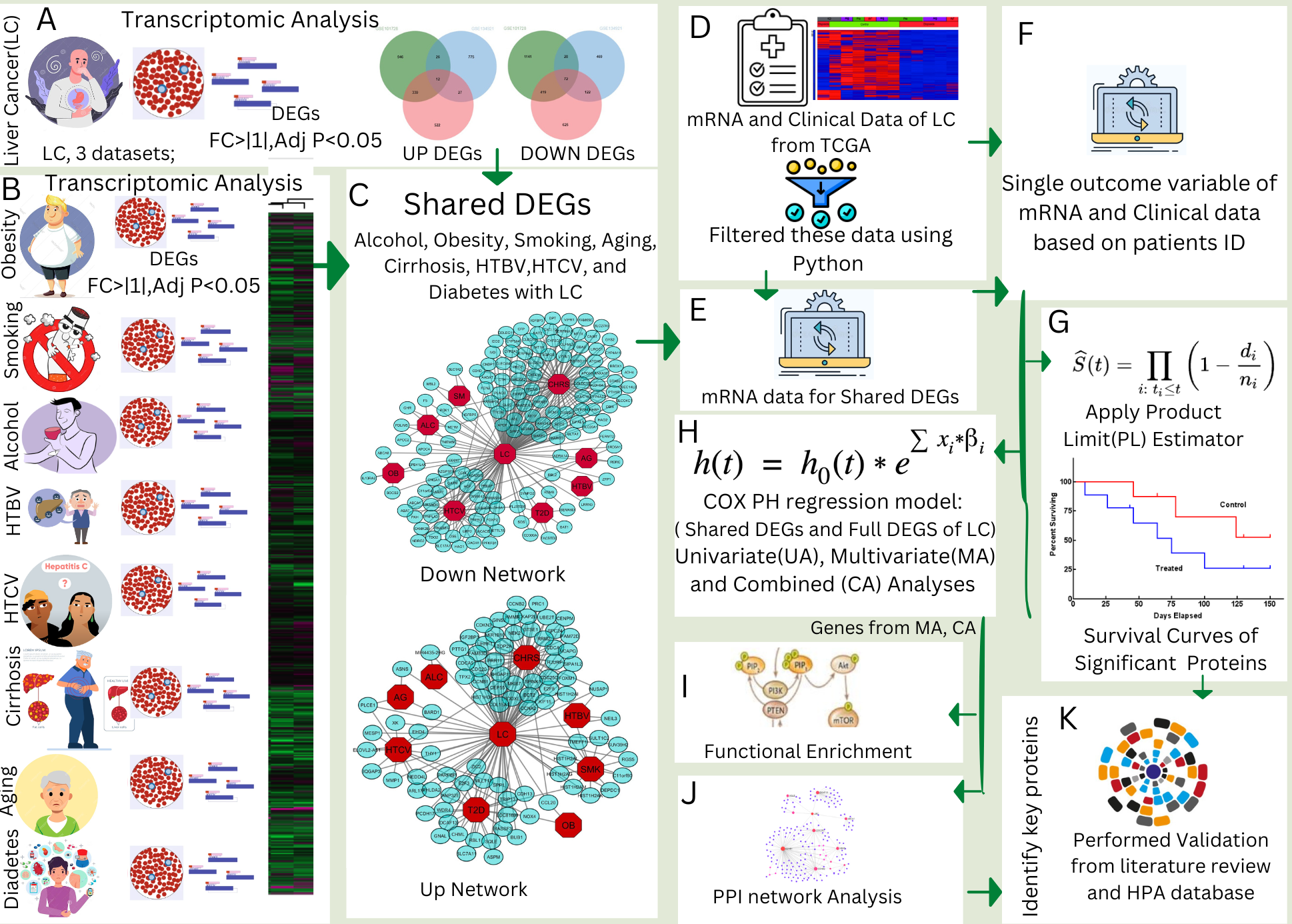

Supplement: Revised-Manuscripts_R2-BFGP-24-0136_elaf019 [file revised-manuscripts_r2-bfgp-24-0136_elaf019.zip › Ali_LC_BIB (1)/LC_WorkF.png]

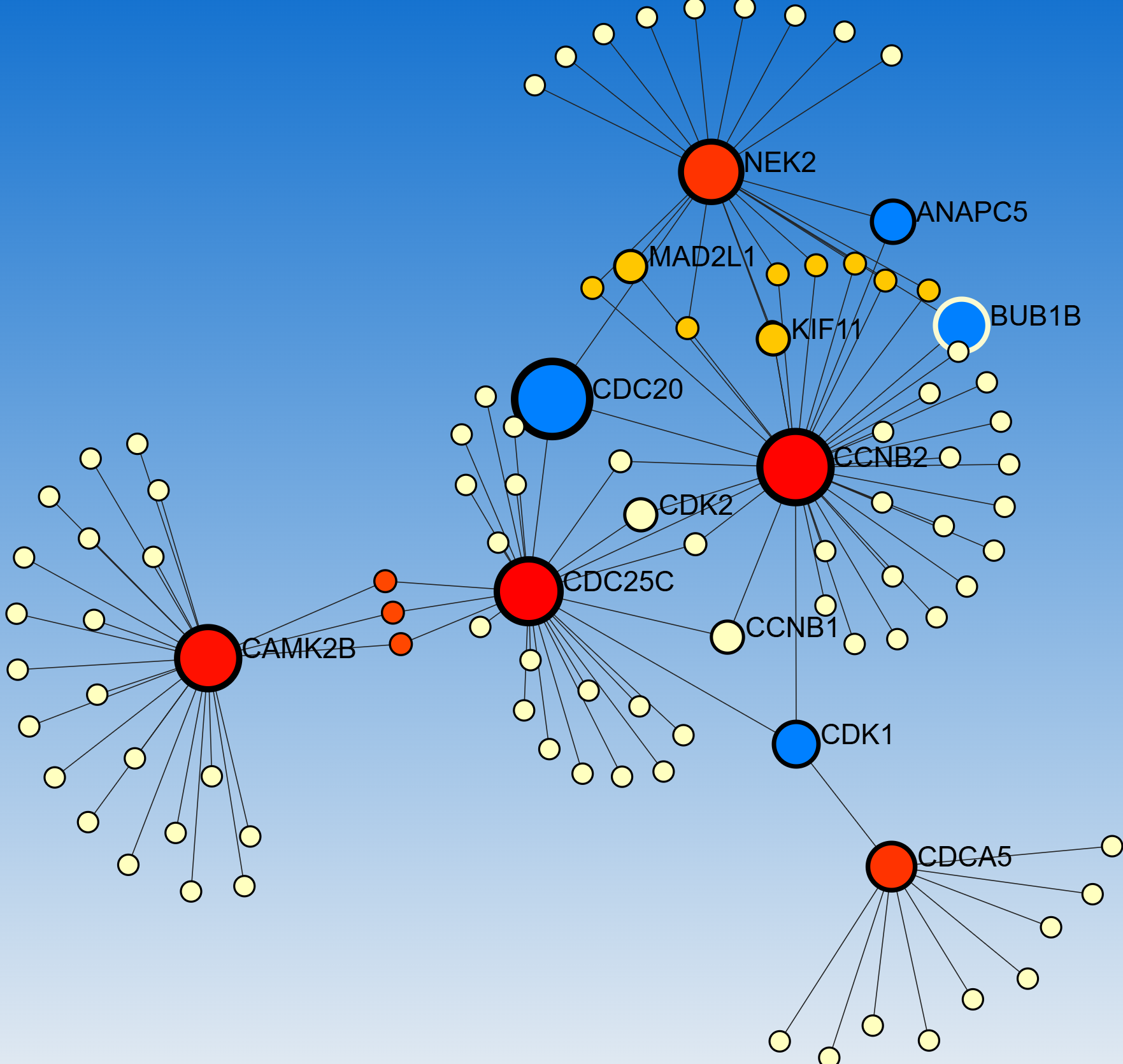

Supplement: Revised-Manuscripts_R2-BFGP-24-0136_elaf019 [file revised-manuscripts_r2-bfgp-24-0136_elaf019.zip › Ali_LC_BIB (1)/PPI2.png]

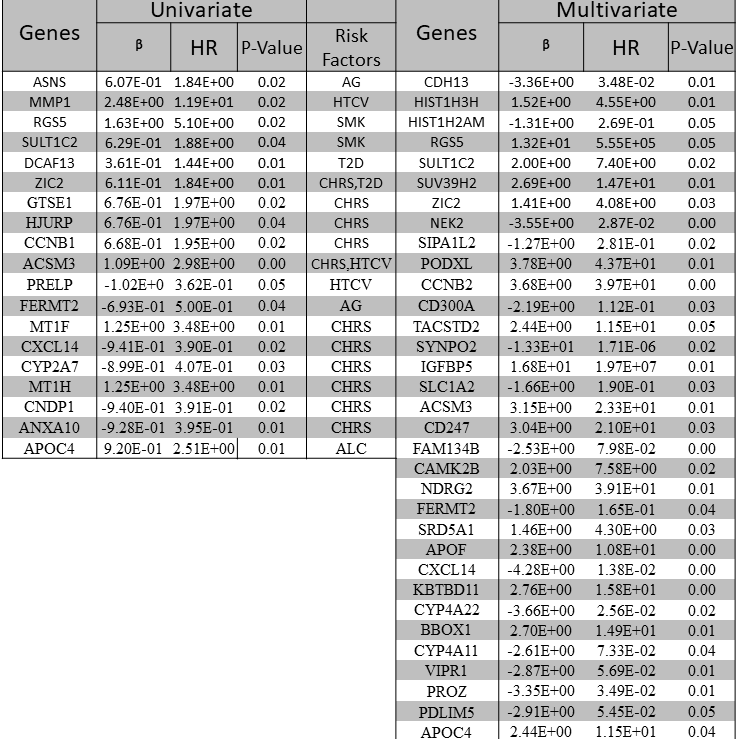

Supplement: Revised-Manuscripts_R2-BFGP-24-0136_elaf019 [file revised-manuscripts_r2-bfgp-24-0136_elaf019.zip › Ali_LC_BIB (1)/SigniGuniMulti.png]

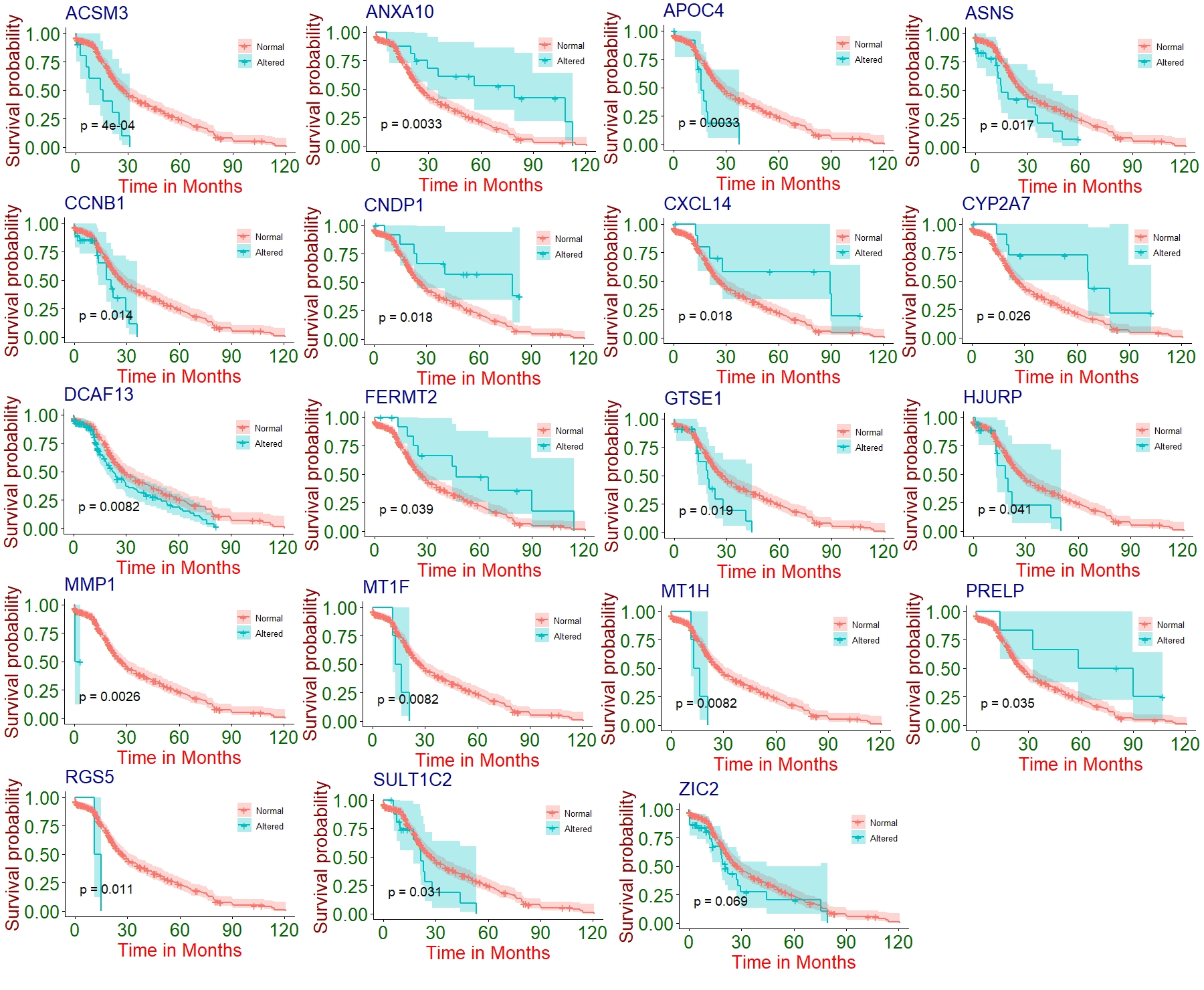

Supplement: Revised-Manuscripts_R2-BFGP-24-0136_elaf019 [file revised-manuscripts_r2-bfgp-24-0136_elaf019.zip › Ali_LC_BIB (1)/Surv.png]

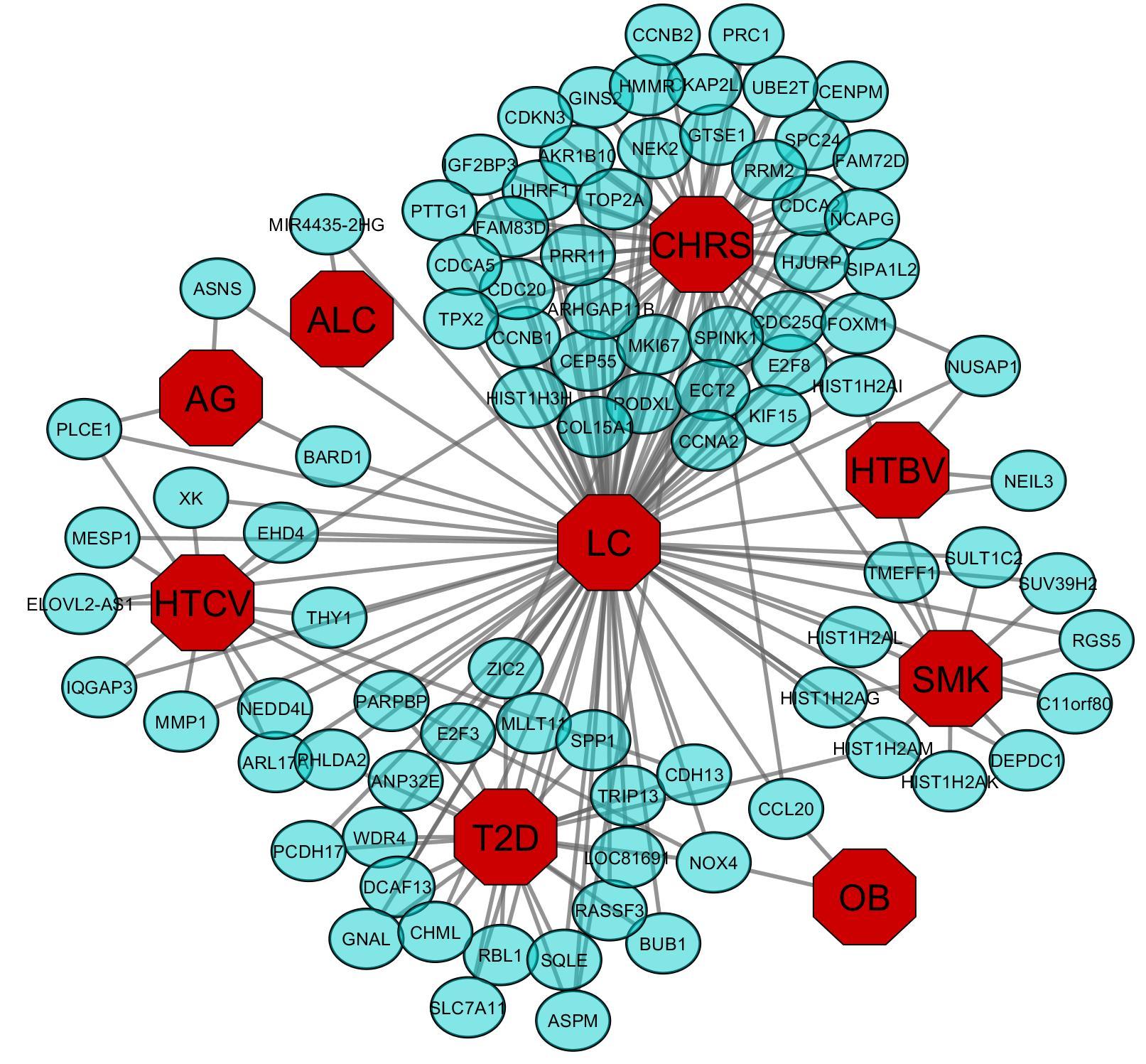

Supplement: Revised-Manuscripts_R2-BFGP-24-0136_elaf019 [file revised-manuscripts_r2-bfgp-24-0136_elaf019.zip › Ali_LC_BIB (1)/UpNetwork.jpeg]

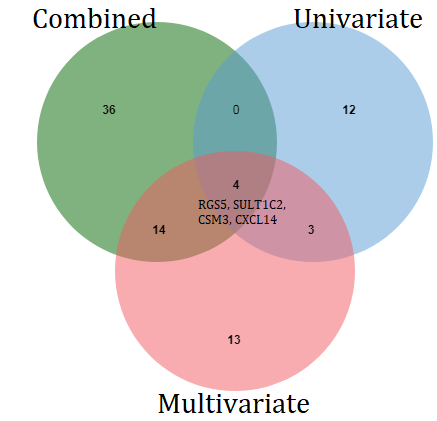

Supplement: Revised-Manuscripts_R2-BFGP-24-0136_elaf019 [file revised-manuscripts_r2-bfgp-24-0136_elaf019.zip › Ali_LC_BIB (1)/vn.png]
